# Estrogen induces St6gal1 expression and increases IgG sialylation in mice and patients with rheumatoid arthritis: a potential explanation for the increased risk of rheumatoid arthritis in postmenopausal women

**DOI:** 10.1186/s13075-018-1586-z

**Published:** 2018-05-02

**Authors:** Cecilia Engdahl, Albert Bondt, Ulrike Harre, Jasmin Raufer, René Pfeifle, Alessandro Camponeschi, Manfred Wuhrer, Michaela Seeling, Inga-Lill Mårtensson, Falk Nimmerjahn, Gerhard Krönke, Hans U. Scherer, Helena Forsblad-d’Elia, Georg Schett

**Affiliations:** 1 Department of Internal Medicine 3, Rheumatology and Immunology, Universitätsklinikum Erlangen, Friedrich Alexander University Erlangen-Nürnberg, Erlangen, Germany; 2 Department of Rheumatology and Inflammation Research, Institute of Medicine, University of Gothenburg, Gothenburg, Sweden; 3 Centre for Bone and Arthritis Research, Department of Internal Medicine and Clinical Nutrition, Institute of Medicine, University of Gothenburg, Gothenburg, Sweden; 4 Department of Rheumatology, Leiden University Medical Center, Leiden, The Netherlands; 5 Center for Proteomics and Metabolomics, Leiden University Medical Center, Leiden, The Netherlands; 6 Institute of Genetics at the Department of Biology, FAU Erlangen-Nuremberg, Erlangen, Germany; 7 Department of Public Health and Clinical Medicine, Umeå University, Umeå, Sweden

**Keywords:** Rheumatoid arthritis, Female sex, Estrogen, Antibody sialylation

## Abstract

**Background:**

Rheumatoid arthritis (RA) preferentially affects women, with the peak incidence coinciding with estrogen decrease in menopause. Estrogen (E2) may therefore have intrinsic immune-regulatory properties that vanish with menopause. Fc sialylation is a crucial factor determining the inflammatory effector function of antibodies. We therefore analyzed whether E2 affects immunoglobulin G (IgG) sialylation.

**Methods:**

Postmenopausal (ovariectomized) mice were immunized with ovalbumin and treated with E2 or vehicle. Total and ovalbumin-specific IgG concentrations, sialylation, and Fcγ receptor expression were analyzed. Postmenopausal women with RA receiving hormone replacement therapy, including E2, or no treatment were analyzed for IgG sialylation. Furthermore, effects of E2 on the expression of the sialylation enzyme β-galactoside α2,6-sialyltransferase 1 (St6Gal1) were studied in mouse and human antibody-producing cells.

**Results:**

E2 treatment significantly increased Fc sialylation of total and ovalbumin-specific IgG in postmenopausal mice. Furthermore, E2 led to increased expression of inhibitory Fcγ receptor IIb on bone marrow leukocytes. Treatment with E2 also increased St6Gal1 expression in mouse and human antibody-producing cells, providing a mechanistic explanation for the increase in IgG-Fc sialylation. In postmenopausal women with RA, treatment with E2 significantly increased the Fc sialylation of IgG.

**Conclusions:**

E2 induces anti-inflammatory effector functions in IgG by inducing St6Gal1 expression in antibody-producing cells and by increasing Fc sialylation. These observations provide a mechanistic explanation for the increased risk of RA in conditions with low estrogen levels such as menopause.

**Electronic supplementary material:**

The online version of this article (10.1186/s13075-018-1586-z) contains supplementary material, which is available to authorized users.

## Background

A person’s gender plays a major role in the development of rheumatoid arthritis (RA). Nearly 75% of patients with RA are women. The reason for the gender imbalance is unclear, but sex hormones are considered to be of pivotal importance. Particularly, the decrease of estrogen in menopause coincides with an increased risk of developing RA [1]. Despite this remarkable association, studies addressing the role of estrogen in the development of RA are scarce [2], and mechanistic studies are virtually absent. Hence, the reason for the preponderance of RA in postmenopausal women remains unclear to date.

RA starts with an autoimmune phase followed by an inflammatory phase [3–5]. Whereas autoimmunity remains clinically silent, inflammation unequivocally leads to symptoms such as pain and swelling. Autoantibodies such as anti-citrullinated peptide antibodies (ACPAs) have a diagnostic, predictive, and prognostic role in RA and can be detected in the preclinical phase several years before the onset of symptoms [6, 7]. These observations indicate that B-cell-mediated autoimmunity and autoantibody development is crucial for the onset of inflammation in RA. Data derived from mouse arthritis models support this concept by showing that B cells, autoantibodies, and Fcγ receptors (FcγRs), which mediate the effector function of autoantibodies, are necessary for the development of arthritis [8–10].

Besides their role in antigen recognition, antibodies regulate effector cell activation through their constant Fc regions, which bind to FcγRs and activate monocytes/macrophages. Antibodies bear one or several carbohydrate chains, or glycans. Glycan at the Asn297 position at the Fc part of IgG regulates binding capability to FcγRs [11–13]. This glycan is composed of a conserved heptamer that consists of N-acetylglucosamine and mannose residues, which can be extended by fucose, galactose, and finally sialic acids. The composition of the IgG-Fc glycosylation, in particular without terminal sialic acids, determines effector cell activation and hence the inflammatory properties of antibodies [14]. Low sialylation of Asn297 enhances the proinflammatory activity [15–19], whereas the attachment of terminal sialic acid residues mediates anti-inflammatory effects [20]. Importantly, it has been shown that the transition from asymptomatic autoimmunity to RA is associated with a change in the sialylation status of antibodies [20, 21].

Population studies have revealed that IgG-Fc galactosylation and sialylation are higher in premenopausal women than in men but decrease with age [22, 23]. During pregnancy, when women are protected from RA, IgG-Fc sialylation as well as galactosylation increase [24, 25], and then reverse within 3 months postdelivery, when RA risk is higher [25]. Estrogen has been shown to decrease galactosylation of human IgG in healthy individuals [26], which may explain the increased risk of RA in postmenopausal women. Whether estrogen influences IgG sialylation has not been investigated yet. In the present study, we show that estrogen influences the presence of sialic acid on the Fc glycan of IgG, both in postmenopausal mice challenged by immunization and in postmenopausal women with RA. This effect is based on estrogen-mediated induction of β-galactoside α2,6-sialyltransferases 1 (St6gal1) in plasmablasts, the enzyme that adds sialic acid residues to IgGs. Hence, decrease in estrogen in menopause creates a proinflammatory state characterized by low IgG sialylation and increased risk of developing arthritis.

## Methods

### Animals and treatments

Female C57BL/6 mice were kept under standard conditions with standard chow and tap water ad libitum. The study was approved by the ethics committees of the Government of Mittelfranken (Germany) and the University of Gothenburg (Sweden). To avoid confounding endogenous sex hormone effects and to mimic a postmenopausal state, mice were ovariectomized (OVX) or sham-operated at 10 weeks of age. Estrogen (17β-estradiol, E2) treatment was carried out with slow-release subcutaneous pellets (Innovative Research of America, Sarasota, FL, USA) containing E2 (0.83 μg/day) or placebo. Treatment of mice with such doses is known to result in serum E2 levels of approximately 60 pg/ml [27]. In mice, normal serum levels of E2 vary between 25 and 50 pg/ml in diestrus and between 150 and 200 pg/ml in estrus [28]. Thus, the dose used in this study resulted in physiological serum E2 levels. Treatment efficiency was confirmed by the weighing of the uterus.

### Immunization with ovalbumin

Mice were subcutaneously immunized with 100 μg of ovalbumin (OVA) (Sigma-Aldrich, St. Louis, MO, USA) emulsified in complete Freund’s adjuvant (Sigma-Aldrich). Followed with a booster injection the same way including 100 μg of OVA (Sigma-Aldrich) emulsified  in incomplete Freund’s adjuvant after 14 days. Serum was taken before OVA immunization, 10 days after initial immunization (day 22), and 10 days after boost (day 38).

### Hormone replacement therapy

Postmenopausal women with RA (*N* = 49) aged 45–65 years were included in a 2-year, randomized, single-blind, controlled study [29]. Patients had active disease with at least two of the following criteria: at least six painful joints, at least three swollen joints, erythrocyte sedimentation rate ≥ 20 mm/h, and C-reactive protein ≥10 mg/L. Patients fulfilled the American Rheumatism Association 1987 criteria for RA [30]. Women in the hormone replacement therapy (HRT) group were given continuous treatment with 2 mg of E2 plus 1 mg of norethisterone acetate daily. All patients provided informed consent, and the ethics committee at the University of Gothenburg (Sweden) approved the study.

### Serum measurements

In the sera from the human HRT study, ACPA were evaluated by enzyme-linked immunosorbent assay (ELISA) (Orgentec Diagnostika, Mainz, Germany). In mice, IgGs were measured using commercially available kits (Bethyl Laboratories, Montgomery, TX, USA). OVA-specific IgG were measured with an in-house ELISA, plates were coated with 100 μg/ml OVA (Sigma-Aldrich), incubated with sera (diluted 1:5000), and detected with horseradish peroxidase (HRP)-conjugated polyclonal rabbit antimouse IgG (Dako/Agilent Technologies, Waldbronn, Germany). For evaluation of the affinity properties of antibodies, potassium thiocyanate (Sigma-Aldrich) was added in various doses. For measurement of sialic acid residues on IgG or OVA-specific IgG, biotinylated *Sambucus nigra* lectin (Vector Laboratories, Burlingame, CA, USA) and streptavidin-HRP (R&D Systems, Minneapolis, MN, USA) were used for detection.

### Isolation of OVA-specific antibodies

OVA-specific antibodies were captured from serum of OVA-immunized mice. Protein G-isolated total IgG was dialyzed in sodium phosphate dibasic and enriched over OVA-coupled Sepharose 4B beads (Sigma-Aldrich), washed with NaCl four times, and eluted with lectin buffer. ELISA confirmed enrichment of OVA-specific IgG.

### Mass spectrometric analysis for Fc glycans

For the analysis of Fc glycosylation, the IgG eluates were subjected to tryptic digestion by adding 600 ng of tosyl phenylalanyl chloromethyl ketone-treated trypsin (Merck, Kenilworth, NJ, USA) in 40 μl of ammonium bicarbonate buffer followed by overnight incubation at 37 °C. Digested IgG was separated and analyzed using a Dionex UltiMate 3000 UHPLC system (Thermo Fisher Scientific, Waltham, MA, USA) coupled to a maXis Impact HD quadrupole-time-of-flight mass spectrometer (MS) (Bruker Daltonics, Billerica, MA, USA). Details are described in Additional file 1: Supplementary methods. The quality of mass spectra was evaluated on the basis of total intensities per glycopeptide cluster. Analyte curation was performed using the signal-to-noise ratio, isotopic pattern quality, and observed mass-to-charge ratio (*m/z*) deviation as obtained after data (pre-)processing with LacyTools [31]. Following extraction of tryptic glycopeptides by a C18 solid-phase extraction trap column (Dionex Acclaim PepMap 100; Thermo Fisher Scientific), separation was performed with a Supelco Ascentis Express C18 nano-LC column (Sigma-Aldrich) conditioned at 900 nl/min with 0.1% trifluoroacetic acid (mobile phase A), after which the following gradient of mobile phase A and 95% acetonitrile (mobile phase B) was applied: 0 minutes 3% B, 2 minutes 6% B, 4.5 minutes 18% B, 5 minutes 30% B, 7 minutes 30% B, 8 minutes 1% B, and 11 minutes 1% B. The ultraperformance LC was interfaced to the MS with a CaptiveSpray electrospray ionization source and nanoBooster (Bruker Daltonics). Mass spectra were recorded from *m/z* 550 to 1800 at a frequency of 1 Hz. Quadrupole ion energy and collision energy of the MS were set at 2 and 5 eV, respectively. The total analysis time per sample was 13 minutes. Detailed calculations are provided in Additional file 1: Supplementary methods.

### Cell preparation and flow cytometry

Cell suspensions were obtained from spleen and bone marrow and stained for surface markers after erythrolysis, fixation, and permeabilization (eBioscience, San Diego, CA, USA). Analyses were performed using a Gallios flow cytometer (Beckman Coulter Life Sciences, Indianapolis, IN, USA) and Kaluza Flow Analysis software (Beckman Coulter Life Sciences). The following fluorochrome- or biotin-conjugated antimouse antibodies and reagents were used: allophycocyanin (APC)-conjugated anti-CD267 (transmembrane activator calcium modulator and cyclophilin ligand interactor), fluorescein isothiocyanate (FITC)-conjugated anti-B220, and FITC-conjugated anti-CD11b (all from eBioscience); phycoerythrin (PE)-conjugated anti-CD138 and PE-cyanine 7 (Cy7) conjugated anti-CD3, Pacific Blue F4/80, PE-Cy7 Ly6G, and PE-FcγRIII (CD16) (all from BioLegend, San Diego, CA, USA); APC-conjugated FcγRI (CD64), FcγRIIB, and FcγRIV (self-made); Alexa Fluor 488-conjugated OVA-A488 (Thermo Fisher Scientific); anti-St6gal1) (C) (IBL International/Tecan, Morrisville, NC, USA); and normal rabbit IgG (isotype-matched control antibody) (Thermo Fisher Scientific).

### Mouse B-cell proliferation

Splenic B cells were isolated by CD43 depletion using MACS technology (Miltenyi Biotec, Bergisch Gladbach, Germany). The cells were stimulated with lipopolysaccharide (LPS) (Sigma-Aldrich) and cultured for 48 hours to develop them into plasmablasts. The medium was then changed to serum-free medium with no estrogen or 10^− 8^ M 17β-estradiol (E2) (Sigma-Aldrich) for the last 24 hours. Blocking antibodies toward IL-22 (Poly5164; BioLegend) and tumor necrosis factor (TNF)-α (Ultra-LEAF anti-TNF-α, MP6-XT22; BioLegend) were added. Seventy-two hours after initial seeding and 24 hours after change in medium, supernatants were collected and cells were isolated for RNA analyses.

### Plasma cell isolation

CD138^+^ splenic plasmablasts were isolated using MACS technology (Miltenyi Biotec) from OVX OVA-immunized mice treated with estrogen or placebo. The purified cells were isolated for RNA analyses.

### Human B-cell proliferation

Human B cells were purified from peripheral blood mononuclear cells using immunomagnetic beads (Dynal® B Cell Negative Isolation Kit; Thermo Fisher Scientific). The cells were stimulated with TLR-9 agonist cytosine-phosphate-guanine (CpG) oligodeoxynucleotide 2006 (InvivoGen, San Diego, CA, USA), goat anti-human IgA/IgG/IgM F(ab′)_2_ fragments (Jackson ImmunoResearch Laboratories, West Grove, PA, USA), and human recombinant IL-2 (R&D Systems), then cultured for 5 days to develop them into plasmablasts. After 5 days, medium was changed to no estrogen or 10^− 8^ M 17β-estradiol (E2) (Sigma-Aldrich). Seven days after initial seeding and 48 hours after medium change, cells were isolated for RNA analyses.

### RT-PCR

Total RNA was extracted using an RNeasy kit (Qiagen) and transcribed into complementary DNA using oligo(dT) primers and MuLV reverse transcriptase (Roche Diagnostics, Indianapolis, IN, USA). qRT-PCR was performed with SYBR Green I dTTP (Eurogentec, Liège, Belgium) or the Applied Biosystems StepOnePlus™ Real-Time PCR system (Thermo Fisher Scientific) using an assays-on-demands primer and probe set. The gene expression values were normalized to those of the control gene encoding β-actin and 18S. Primer sequences are described in Additional file 1: Supplementary methods.

### Statistical analysis

Statistical analyses were performed using Prism software (GraphPad Software, La Jolla, CA, USA). Two separate groups were compared with unpaired Student’s *t* tests. One-way analysis of variance followed by Bonferroni multiple-comparisons tests were performed for selected columns. Outliers were eliminated using Grubbs’ test. The correlation was investigated with Spearman’s correlation coefficients. Data are presented as mean ± SEM or as scatterplots, and *p* < 0.05 was considered significant.

## Results

### Effects of menopausal state and E2 treatment on antibody responses to ovalbumin immunization

**Fig. 1:**
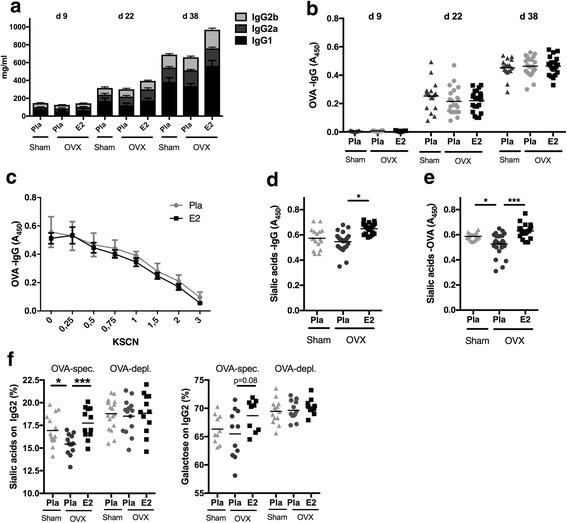
Estrogen influences immunoglobulin G (IgG) sialylation. Mice were ovariectomized (OVX) at 3 months of age, followed by insertion of slow-release treatment pellets with placebo (Pla) or estrogen (E2; 0.83 μg/day). Ten days after ovariectomy, the animals were immunized with ovalbumin (OVA), and 14 days later, they were boostered. Serum was taken at day 9 (d9) before immunization, day 22 (d22) after immunization, and day 38 (d38) at termination after immunization and boostering. **a** Total IgG concentrations: significant induction in all treatment groups after immunization (day 9–day 22) and after boostering (day 22–day 38) for all IgG subtypes. Data are mean ± SEM (*n* = 11–13). **b** Concentration of ovalbumin-specific IgG (OVA-IgG) (*n* = 17–24). **c** Titers of ovalbumin-specific IgG measured at termination (*n* = 10–12). **d** Concentration of sialic acids on total IgG measured at termination (n = 17–24). **e** Concentration of sialic acids on OVA-specific IgG measured at termination (*n* = 17–24). **f** Mass spectrometry-based analysis of sialic acids and galactose on IgG-Fc of OVA-specific (OVA-spec) and OVA-depleted (OVA-dep) IgG2 measured at termination (*n* = 12–14). Values are indicated on a scatter dot plot with mean indicated by a bar. Statistical analysis with analysis of variance followed by the Bonferroni multiple-comparisons test for selected time points. *p < 0.05, ***p < 0.001

To understand the effect of estrogen on the humoral immune response, we immunized mice with OVA. Prior to immunization, mice were either sham-operated or OVX with or without subsequent treatment with E2, reflecting HRT or estrogen deficiency, respectively. As expected, OVA immunization increased total IgG and OVA-specific IgG (Fig. 1a and b). E2 treatment increased total IgG2b and IgG1 levels, but no differences were found in the development of OVA-specific IgG, suggesting that E2 does not influence antibody titers after immunization. Also, the affinity of OVA-specific antibodies was not affected by estrogen status (Fig. 1c).

### Effects of menopausal state and E2 treatment on IgG sialylation

Estrogen treatment increased terminal sialic acid residues of total IgG (Fig. 1d). Even more interesting, this increase was also seen in OVA-specific IgG (Fig. 1e), whereas ovariectomy itself induced a significant decrease of sialylation of OVA-specific IgG. Mass spectrometric analysis of purified OVA-specific IgG2 showed significantly lower sialic acid content in OVX mice, whereas E2 treatment rescued IgG2 sialylation (Fig. 1f). In contrast, no difference was found in OVA-depleted IgG2, indicating that estrogen determines the sialylation of newly formed antigen-specific antibodies. Galactose, which is positioned proximal to sialic acids was much less affected by E2 (Fig. 1f), although a trend (*p* = 0.08) toward higher galactosylation was found in OVA-specific IgG2 after E2 treatment. Overall, these results indicate that estrogen status regulates the sialic acid content of newly formed IgG. Conversely, E2 deficiency suppresses the sialylation of IgGs. These observations support the hypothesis that E2 could regulate IgG-mediated effector functions.

### Effects of menopausal state and E2 treatment on FcγR expression

**Fig. 2:**
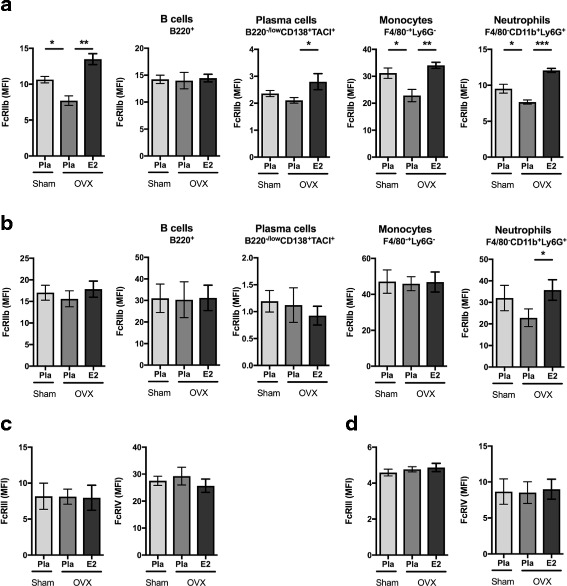
Effects of estrogen on Fcγ receptor expression. Ovariectomized (OVX) mice at 3 months of age followed by insertion of slow-release treatment pellets with placebo (Pla) or estrogen (E2; 0.83 μg/day). Ten days after ovariectomy, the animals were immunized with ovalbumin (OVA), and 14 days later, they were boostered. On day 38, mean fluorescence intensity (MFI) of Fc receptors (FcRs) was quantified using flow cytometry in all leukocytes as well as B cells, plasmablasts, monocytes, and neutrophils. Expression of inhibitory FcγRIIb in (**a**) bone marrow cells and (**b**) spleen cells. Expression of stimulatory FcγRIII and FcγRIV in (**c**) bone marrow cells and (**d**) spleen cells. Groups are presented with a bar indicating mean ± SEM (*n* = 6–7). Statistical analysis with analysis of variance followed by the Bonferroni multiple-comparisons test for selected time points. *p < 0.05, **p < 0.01, ***p < 0.001

We next determined estrogen regulation of FcγR expression in OVA-challenged mice treated with E2. E2 status affects B-cell and plasma cell frequency; therefore, the mean fluorescence intensity was used to determine the expression quantity of each FcγR. We observed that in the bone marrow, estrogen significantly increased the expression of the inhibitory FcγRIIb in plasmablasts, monocytes, and neutrophils (Fig. 2a). In the spleen, similar effects could be observed only with neutrophils (Fig. 2b). Expression of activating FcγRIII and FcγRIV was not affected by either ovariectomy or E2 treatment (Fig. 2c and d). Furthermore, OVA-specific IgG subclass composition was not significantly affected by ovariectomy or E2 treatment, although a trend toward lower IgG2a and IgG2b levels was found after ovariectomy (Additional file 2: Supplementary Figure 1).

### E2 increases the expression of β-galactoside α2,6-sialyltransferase 1 in plasmablasts

**Fig. 3:**
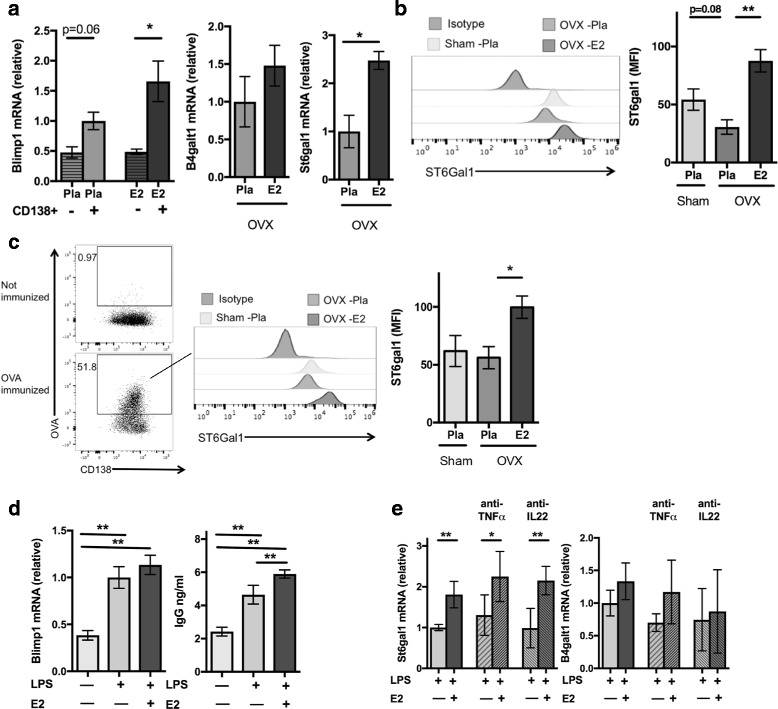
Estrogen affects expression of the sialylation enzyme in plasmablasts. Mice were ovariectomized (OVX) at 3 months of age, followed by insertion of slow-release treatment pellets with placebo (Pla) or estrogen (E2; 0.83 μg/day). Ten days after ovariectomy, animals were immunized with ovalbumin (OVA), and 14 days later, they were boostered. **a** Expression of Blimp1, β-1,4-galactosyltransferase 1 (B4galt1), and β-galactoside α2,6-sialyltransferase 1 (St6gal1) in CD138-sorted plasmablasts on day 38 (*n* = 5–6). Statistical analysis was performed with Student’s *t* test. **b** Flow cytometry-based quantification of mean fluorescence intensity (MFI) of St6Gal1 expression of plasmablasts on day 38 (*n* = 10–14). Statistical analysis was performed with analysis of variance (ANOVA) followed by the Bonferroni multiple-comparisons test. **c** Flow cytometry-based quantification of MFI St6Gal1 expression of OVA-specific plasmablasts (*n* = 10–14). Statistical analysis was performed with ANOVA followed by the Bonferroni multiple-comparisons test. **d** Immunoglobulin G in supernatant and expression of Blimp1, in CD43-negative splenic cells stimulated with lipopolysaccharide (LPS) for 72 hours for development into plasmablasts. For the last 24 hours, the cells were stimulated with the presence (+) of estrogen (E2) 10^− 8^ or absence (−) of E2. **e** Expression of B4galt1, and St6gal1 in CD43-negative splenic cells stimulated with lipopolysaccharide (LPS) for 72 h for development into plasmablasts. For the last 24 h, the cells were stimulated with the presence (+) of estrogen (E2) 10− 8 or absence (−) of E2. Anti-tumor necrosis factor (TNF)-α or ant-interleukin (IL)-22 antibodies were also added as indicated. Statistical analysis was performed with Student’s *t* test (*n* = 5–7). *p < 0.05, **p < 0.01. Data are shown as mean ± SEM. *mRNA* Messenger RNA

To explain the effects of E2 on IgG Fc sialylation, we analyzed the expression of St6gal1, the enzyme responsible for the attachment of sialic acid residues to IgG, in plasmablasts. mRNA was isolated from splenic plasmablasts purified from postmenopausal mice (OVX) with and without E2 treatment that had been challenged by OVA immunization. The identity of the plasmablasts was confirmed by measuring mRNA expression of the plasma cell-specific marker Blimp1 (Fig. 3a). Expression of St6gal1 was significantly upregulated by E2. In contrast, expression of β-1,4-galactosyltransferase 1 (B4galt1), which is responsible for IgG galactosylation, was hardly affected. St6gal1 protein expression of plasmablasts was further investigated using flow cytometric analysis. E2 treatment showed significantly higher expression of St6gal1 in total splenic plasmablasts (Fig. 3b) as well as in OVA-specific plasmablasts (Fig. 3c).

To determine whether E2 directly affects St6gal1 expression or whether this effect is mediated by cytokines, we stimulated naive splenic B cells by LPS to induce plasmablast differentiation. Blimp1 mRNA was upregulated and IgG production was increased, confirming plasmablast differentiation (Fig. 3d). E2 treatment upregulated IgG levels compared with E2 restriction. As expected, St6gal1 mRNA was significantly upregulated upon E2 treatment, whereas no effect was seen for B4galt1 (Fig. 3e). Cytokines such as TNF-α, which is regulated by E2 [32], as well as IL-22, have been shown to influence IgG glycosylation [20, 33]. We therefore blocked TNF-α and IL-22 to test whether cytokine inhibition influences the effect of E2 on ST6gal1 expression. However, upregulation of St6gal1 was still evident after blocking with anti-TNF-α and anti-IL-22 antibodies (Fig. 3e), suggesting that E2 upregulation of St6gal1 is independent of TNF-α and IL-22.

### E2 treatment in patients with RA increases antibody sialylation

**Fig. 4:**
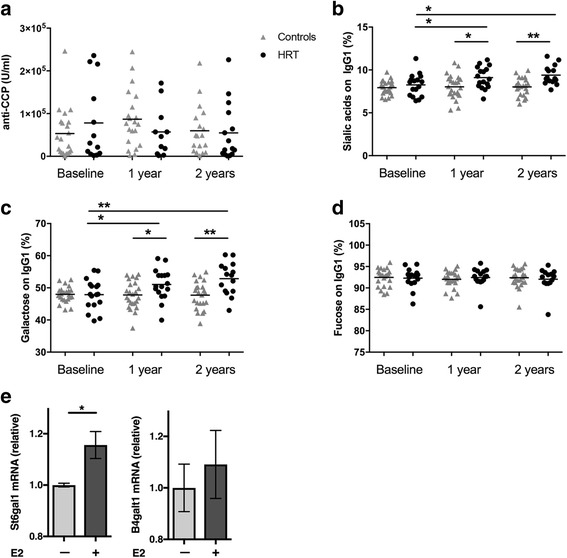
Estrogen increases immunoglobulin G (IgG) glycosylation in postmenopausal patients with rheumatoid arthritis (RA). Forty-nine postmenopausal women with active RA in a randomized controlled trial were receiving either hormone replacement therapy (HRT) or no HRT. Serum samples were taken at baseline and after 1 year and 2 years of treatment. **a** Anticitrullinated protein antibody concentrations (*n* = 20–29; 7 anti-cyclic citrullinated peptide [anti-CCP]-negative patients were excluded from further studies). Mass spectrometry-based analysis of (**b**) sialic acids, (**c**) galactose, and (**d**) fucose on the Fc portion of IgG. Statistical analysis was performed with *t* tests at selected time points, as well as an investigation over time in HRT-treated individuals. *p < 0.05, **p < 0.01 (*n* = 17–25). Groups are presented with a bar indicating mean with SEM. **e** Messenger RNA (mRNA) expression of St6gal1 and B4galt1 from B cells isolated from peripheral blood collected from healthy donors and stimulated with CpG, interleukin-2, and goat antihuman IgA/IgG/IgM F(ab′)_2_ fragments for 5 days to induce plasmablast differentiation. For the last 48 hours, the cells were stimulated with the presence (+) of estrogen (E2) 10^− 8^ or absence (−) of E2. Statistical analysis was performed with Student’s t test. *p < 0.05 (*n* = 3). Values are indicated as scatter dot plots with means indicated with a bar

To confirm the relevance of the above-described findings for patients with RA, we examined serum samples of 49 postmenopausal women with RA treated with or without HRT [29, 34, 35]. In a previous trial, HRT treatment was shown to reduce disease activity of RA and to increase bone mineral density. On the basis of a total of seven ACPA-negative patients (three in the HRT-treated group and four in the control group), there was no difference in ACPA titers between patients receiving HRT or not receiving HRT (Fig. 4a). For the next investigations, we concentrated on ACPA-positive patients and found that IgG galactosylation and sialylation in the 42 ACPA-positive patients were increased in the HRT-treated patients in both the Fc regions of IgG1 (Fig. 4b and c) and IgG2/3 (Additional file 3: Supplementary Figure 2a and b). Fucose residues were not altered in the Fc regions of IgG1 (Fig. 4d) or IgG2/3 (Additional file 3: Supplementary Figure 2c). These data indicate that E2 treatment increases sialic acid content on IgG-Fc tails in postmenopausal women with RA. The increase in IgG sialylation and galactosylation in individual patients over time is depicted in Additional file 4: Supplementary Figure 3. Furthermore, when correlating the degree of IgG sialylation and galactosylation to serum E2 levels, we found a significant positive correlation, adding further evidence that E2 can regulate the effector function of IgGs in humans (Additional file 5: Supplementary Figure 4a and b). In addition, IgG sialylation and galactosylation showed a significant inverse correlation with RA disease activity as measured by Disease Activity Score [29] (Additional file 5: Supplementary Figure 4c and d).

### E2 increases St6gal1 expression in human plasmablasts

Finally, and on the basis of data obtained from the human cohort, we analyzed whether E2 can induce St6gal1 expression in human plasmablasts. To test this, we isolated human B cells from healthy control subjects, differentiated them into plasmablasts, and added E2 for the last 48 hours. E2 significantly upregulated mRNA expression of St6gal1 in the human plasmablasts, whereas no such effect was observed for B4galt1 (Fig. 4e). Hence, E2 also induces St6gal1 and IgG sialylation in humans.

## Discussion

In the present study, we show that E2 influences IgG glycosylation, especially the sialylation of IgG, by upregulating the key enzyme St6gal1 in plasmablasts. Deficiency of E2, like in menopause, leads to decreased antibody sialylation and to a proinflammatory IgG pattern, which could influence the onset of RA and may explain the increased risk of RA in postmenopausal women. However, E2 administration increased sialylation of IgG, shifting the antibody effector function to a more regulatory anti-inflammatory pattern, which is supported by a negative correlation between the degree of IgG sialylation and galactosylation with RA disease activity.

In RA, it is well established that autoantibody formation precedes the symptomatic inflammatory phase of the disease. Factors that shift asymptomatic autoimmunity to inflammation are therefore of key interest in understanding the onset of disease [36]. The sialylation status of autoantibodies seems to play a crucial role in this shift. Low-level IgG sialylation promotes progression to inflammation, whereas high-level sialylation promotes suppression of symptoms [20]. Our results indicate that estrogen affects the pathogenicity of the antibodies, mainly via regulation of IgG-Fc sialylation. Hence, higher levels of E2 create an anti-inflammatory environment by inducing St6gal1, resulting in a higher degree of antibody sialylation. In accordance, the sharp decrease of estrogens in menopause is supposed to switch this environment to low sialylation and a proinflammatory pattern.

In contrast to the findings regarding sialylation, we did not detect any significant effect of E2 on galactosylation in mice or on the expression of B4galt1, the enzyme mediating galactosylation, in mouse and human antibody-producing cells. Nonetheless, in postmenopausal patients with RA, treatment with E2 increased not only sialylation but also galactosylation of IgG. Similarly, we observed strong correlations of IgG-Fc galactosylation and levels of estrogen in the postmenopausal patients with RA. These findings are in accordance with previous results in healthy individuals showing that E2 regulates galactosylation [26]. Because galactosylation is a prerequisite for sialylation at the Asn297 site of IgG, an effect of E2 on galactosylation might further strengthen the overall E2-induced glycosylation pattern of human IgG.

E2 effects on B-cell [37–39] and plasma cell differentiation [40, 41] have been reported previously, but functional consequences on the pattern of plasma cell-mediated antibody production have so far been undetermined. On one hand, regulation of St6gal1 by E2 suggests that the overall effect of E2 on the effector pathways of adaptive immunity is a regulatory one and that loss of E2 induces a proinflammatory environment by altering effector functions of antibodies. On the other hand, E2 did not have any consistent effect on specific antibody levels and affinity, suggesting that the key factor by which E2 regulates inflammatory responses is indeed its influence on IgG glycosylation. Future studies need to be done to test whether estrogen treatment in postmenopausal women stimulates St6gal1 in B cells and plasmablasts.

## Conclusions

The data derived from the present study provide a basis for a molecular concept that could explain why susceptibility to RA changes during a woman’s life and specifically increases in menopause. E2 appears to be a protective factor rather than a risk factor in triggering inflammation in arthritis by inhibiting the proinflammatory effector functions of autoantibodies. Higher rates of flares of RA with the decrease of sex hormones after pregnancy [42], as well as the accumulation of flares in the second low-estrogen phase of the menstrual cycle [43], also support this concept. Treatment with E2 may therefore have a beneficial effect in some patients with RA, particularly in those with imminent RA displaying autoantibodies and initial symptoms such as pain, with a high risk of progressing to clinical RA.

## Additional files

Additional file 1: Supplementary methods. (DOCX 131 kb)

Additional file 2: **Supplementary Figure 1.** Estrogen effects on ovalbumin-specific IgG subclasses. Ovariectomized mice received slow-release treatment pellets with placebo (Pla) or estrogen (E2; 0.83 μg/day). Ten days after ovariectomy, the animals were immunized with ovalbumin (OVA), and 14 days later, they were boostered. On day 38, IgG subclasses of ovalbumin (OVA)-specific IgG were measured. A scatterplot with means indicated by lines is shown (*n* = 9–11 mice per group). Statistical analysis was performed with analysis of variance followed by the Bonferroni multiple-comparisons test. (PDF 1088 kb)

Additional file 3: **Supplementary Figure 2.** Estrogen treatment increases IgG-Fc sialylation and IgG-Fc galactosylation in postmenopausal patients with rheumatoid arthritis (RA). Forty-nine postmenopausal women with active RA were included in a randomized controlled trial receiving either hormone replacement therapy (HRT) or no HRT. Serum samples were taken at baseline and after 1 year and 2 years of treatment. Mass spectrometry-based analysis of (**a**) sialic acids, (**b**) galactose, and (**c**) fucose of the Fc portion of IgG2/3 (*n* = 17–25). Statistical analysis was performed with *t* tests at selected time points, as well as in an investigation over time in individuals who received HRT. * *p* < 0.05, *** *p* < 0.001. (PDF 1915 kb)

Additional file 4: **Supplementary Figure 3.** Effects of estrogen on IgG-Fc sialylation and IgG-Fc galactosylation in individual patients. Analysis of (**a**, **b**) IgG-Fc sialylation and (**c**, **d**) IgG-Fc galactosylation in postmenopausal women with active rheumatoid arthritis (RA) randomized to receive hormone replacement therapy (HRT) (**a**, **c**) or no HRT (**b**, **d**) in a controlled trial. Each line represents one patient. (PDF 2784 kb)

Additional file 5: **Supplementary Figure 4.** Correlation between IgG glycosylation and estrogen levels and disease activity. Correlation between (**a**) IgG-Fc sialylation and (**b**) IgG-Fc galactosylation (*y*-axis) and estrogen level (*x*-axis). Correlation between (**c**) IgG-Fc sialylation and (**d**) IgG-Fc galactosylation (*y*-axis) and Disease Activity Score (DAS) (*x*-axis). Spearman’s correlation coefficients (*r*) and *p* values are shown. (PDF 2188 kb)

## Abbreviations

ACPA: Anticitrullinated peptide antibodies
APC: Allophycocyanin
B4galt1: β-1,4-Galactosyltransferase 1
Cy7: Cyanine 7
E2: Estrogen
ELISA: Enzyme-linked immunosorbent assay
FcγR: Fcγ receptor
FITC: Fluorescein isothiocyanate
HRP: Horseradish peroxidase
HRT: Hormone replacement therapy
LPS: Lipopolysaccharide
MS: Mass spectrometer
*m/z*: Mass-to-charge ratio
OVA: Ovalbumin
OVX: Ovariectomized
PE: Phycoerythrin
RA: Rheumatoid arthritis
St6gal1: β-Galactoside α2,6-sialyltransferase 1
TNF: Tumor necrosis factor
