# Supplementary material for: Estrogen induces St6gal1 expression and increases IgG sialylation in mice and patients with rheumatoid arthritis: a potential explanation for the increased risk of rheumatoid arthritis in postmenopausal women
Source: Arthritis Res Ther. 2018 May 2;20:84. doi: 10.1186/s13075-018-1586-z (PMC5932893; doi:10.1186/s13075-018-1586-z)
Supplement: Supplementary file 1 — Supplementary methods. (DOCX 131 kb) [file 13075_2018_1586_MOESM1_ESM.docx]

Estrogen induces St6gal 1 expression and increases IgG sialylation in mice and patients with rheumatoid arthritis- a potential explanation for the increased risk of rheumatoid arthritis in postmenopausal women

**Running head:** Estrogen effects on IgG sialylation

Cecilia Engdahl^1,2,3^, Albert Bondt^4,5^, Ulrike Harre^1^, Jasmin Raufer^1^, René Pfeifle^1^, Alessandro Camponeschi^2^, Manfred Wuhrer^5^, Michaela Seeling^5^, Inga-Lill Mårtensson-Bopp^2^, Falk Nimmerjahn^6^, Gerhard Krönke^1^, Hans U Scherer^4^, Helena Forsblad-d’Elia^2,7^, Georg Schett^1^

^1^Department of Internal Medicine 3, Rheumatology and Immunology, Universitätsklinikum Erlangen, Friedrich Alexander University Erlangen-Nürnberg, Erlangen, Germany, ^2^Department of Rheumatology and Inflammation Research and ^3^Department of Internal Medicine, Institute of Medicine, University of Gothenburg, Gothenburg, Sweden, ^4^Department of Rheumatology, Leiden University Medical Center, Leiden, The Netherlands, ^5^Center for Proteomics and Metabolomics, Leiden University Medical Center, Leiden, The Netherlands ^6^Institute of Genetics at the Department of Biology, FAU Erlangen-Nuremberg, Erlangen, Germany, ^7^Department of Public Health and Clinical Medicine, Umeå University, Umeå, Sweden.

**Email addresses to authors:**

Cecilia Engdahl: [cecilia.engdahl@ gu.se](mailto:cecilia.engdahl@rheuma.gu.se)

Alber Bondt: A.Bondt@lumc.nl

Ulrike Harre; [Ulrike.Harre@uk-erlangen.de](mailto:Ulrike.Harre@uk-erlangen.de)

Jasmin Raufer; [jasmin.raufer@uk-erlangen.de](mailto:jasmin.raufer@uk-erlangen.de)

René Pfeifle: [rene.pfeifle@uk-erlangen.de](mailto:rene.pfeifle@uk-erlangen.de)

Alessandro Camponeschi; [alessandro.camponeschi@rheuma.gu.se](mailto:alessandro.camponeschi@rheuma.gu.se)

Manfred Wuhrer; [m.wuhrer@lumc.nl](mailto:m.wuhrer@lumc.nl)

Michaela Seeling; [michaela.seeling@fau.de](mailto:michaela.seeling@fau.de)

Inga-Lill Mårtensson-Bopp; [lill.martensson@rheuma.gu.se](mailto:lill.martensson@rheuma.gu.se)

Falk Nimmerjahn; [falk.nimmerjahn@fau.de](mailto:falk.nimmerjahn@fau.de)

Gerhard Krönke; [gerhard.kroenke@uk-erlangen.de](mailto:gerhard.kroenke@uk-erlangen.de)

Hans U Scherer4: [H.U.Scherer@lumc.nl](mailto:H.U.Scherer@lumc.nl)

Helena Forsblad-d’Elia; [helena.forsblad@rheuma.gu.se](mailto:helena.forsblad@rheuma.gu.se)

Georg Schett; [georg.schett@uk-erlangen.de](mailto:georg.schett@uk-erlangen.de)

**Corresponding author:** Cecilia Engdahl, PhD or Georg Schett, MD; Department of Internal Medicine 3, Rheumatology and Immunology, Universitätsklinikum Erlangen, Friedrich Alexander University Erlangen-Nürnberg, Erlangen, Ulmenweg 18; 91054 Erlangen, Germany. **E-mail:** cecilia.engdahl@gu.se or [georg.schett@uk-erlangen.de](mailto:georg.schett@uk-erlangen.de) **Phone:** +46 31 342 6427 or +49 91318539193

Abstract

**Background**. Rheumatoid arthritis (RA) preferentially affects women, with the peak incidence coinciding with estrogen decrease in the menopause. Estrogen (E2) may, therefore, have intrinsic immune-regulatory properties that vanishes with menopause. Fc-sialylation is a crucial factor determining the inflammatory effector function of antibodies. We, therefore, analyzed whether E2 affects IgG sialylation.

**Methods**. Postmenopausal (ovariectomized) mice were immunized with ovalbumin and treated with E2 or vehicle. Total and ovalbumin-specific IgG concentrations, sialylation and Fc gamma receptor expression were analyzed. Postmenopausal women with RA receiving hormone replacement therapy, including E2, or no treatment were analyzed for IgG sialylation. Furthermore, effects of E2 on the expression of the sialylation enzyme β-galactoside α2,6-sialyltransferases (St6Gal1) were studied in mouse and human antibody producing cells.

**Results**. E2 treatment significantly increased Fc-sialylation of total and ovalbumin-specific IgG in postmenopausal mice. Furthermore, E2 led to increased expression of inhibitory Fc gamma receptor IIb on bone marrow leucocytes. Treatment with E2 also increased St6Gal1 expression in mouse and human antibody producing cells providing a mechanistic explanation for the increase in IgG Fc-sialylation. In postmenopausal women with RA, treatment with E2 significantly increased the Fc-sialylation of IgG.

**Conclusion**. E2 induces anti-inflammatory effector functions in IgG by inducing St6Gal1 expression in antibody producing cells and by increasing Fc-sialylation. These observations provide a mechanistic explanation for the increased risk of RA in conditions with low estrogen levels such as the menopause.

**Key words:** rheumatoid arthritis, female sex, estrogen, antibody sialylation

Background

A person’s gender plays a major role in the development of rheumatoid arthritis (RA). Hence, nearly 75% of patients suffering from RA are women. The reason for the gender imbalance is unclear but sex hormones are considered being of pivotal importance. Particularly, the decrease of estrogen in the menopause coincides with an increased risk to develop RA,[1]. Despite this remarkable association, studies addressing the role of estrogen in the development of RA are scarce,[2] and mechanistic studies are virtually absent. Hence, the reason for the preponderance of RA in postmenopausal women remains unclear to date.

RA starts with an autoimmune phase followed by an inflammatory phase,[3-5]. While autoimmunity remains clinically silent, inflammation unequivocally leads to symptoms such as pain and swelling. Autoantibodies, like anti-citrullinated peptide antibodies (ACPAs), have a diagnostic, predictive and prognostic role in RA and can be detected in the preclinical phase several years before the onset of symptoms,[6, 7]. These observations indicate that B cell mediated autoimmunity and autoantibody development is crucial for the onset of inflammation in RA. Data from mouse arthritis models support this concept showing that B cells, autoantibodies and Fc gamma receptors (FcγR), which mediate the effector function of autoantibodies, are necessary for the development of arthritis,[8-10].

Besides their role in antigen recognition, antibodies regulate effector cell activation through their constant Fc regions which bind to FcγR and activate monocytes/macrophages. Antibodies bear one or several carbohydrate chains, or glycans. Glycans postion at Asn297 at the Fc part of the IgG regulates binding capability to FcγR,[11-13]. This glycan is composed of a conserved heptamer that consists of N-acetylglucosamine and mannose residues, which can be extended by fucose, galactose and finally sialic acids. The composition of the IgG-Fc glycosylation, in particular those without terminal sialic acids, determines effector cell activation and hence the inflammatory properties of antibodies,[14]. Low sialylation of Asn297 enhances their pro-inflammatory activity,[15-19], while the attachment of terminal sialic acids residues mediates anti-inflammatory effects,[20]. Importantly, it has been shown that the transition from asymptomatic autoimmunity to RA is associated with a change in the sialylation status of antibodies,[20, 21].

Population studies have revealed that IgG-Fc galactosylation and sialylation is higher in premenopausal women compared to men, while decreasing with age,[22, 23]. During pregnancy, when women are protected from RA, IgG-Fc sialylation but also galactosylation increase,[24, 25] and reverse within 3 months post-delivery, when RA risk is higher,[25]. Estrogen has been shown to decrease galactosylation of human IgG in healthy individuals,[26], which may explain the increased risk of RA in postmenopausal women. If estrogen influences IgG sialylation has not been investigated yet. Herein we show that estrogen influences the presence of sialic acid on the Fc glycan of IgG, both in postmenopausal mice challenged by immunization and in postmenopausal women with RA. This effect is based on estrogen-mediated induction of β-galactoside α2,6-sialyltransferases 1 (St6gal1) in plasmablasts, the enzyme that adds sialic acid residues to IgGs. Hence, decrease in estrogen in the menopause creates a proinflammatory state characterized by low IgG sialylation and increased risk to develop arthritis.

Materials and Methods

Animals and treatments

Female C57BL/l6 mice were kept under standard conditions with standard chow and tap water ad libitum. The ethics committees (Government of Mittelfranken, Germany and University of Gothenburg, Sweden) approved these studies. To avoid confounding endogenous sex hormone effects and to mimic a postmenopausal state, mice were ovariectomized (OVX) or sham operated at 10 weeks of age. E2 treatment was done with slow-release subcutaneous pellets (Innovative Research of America) containing 17β-estradiol (E2) (0.83 μg/day) or placebo. Treatment of mice with such doses is known to result in serum E2 levels of approximately 60 pg/m,[27]. In mice, normal serum levels of E2 vary between 25 and 50 pg/mL in diestrus and between 150 and 200 pg/mL in estrus,[28]. Thus, the dose used in this study resulted in physiological serum E2 levels. Treatment efficiency was confirmed by the weighing of the uterus.

Immunization with ovalbumin

Mice were subcutaneously immunized with 100 µg ovalbumin (OVA) (Sigma) emulsified in Freund’s complete adjuvant (Sigma-Aldrich). Mice were boosted with 100 µg OVA in incomplete Freund’s adjuvant after 14 days. 100 μL was injected intradermally at the base of the tail. Serum was taken before OVA immunization, 10 days after initial immunization (day 22) and 10 days after boost (day 38).

Hormone replacement therapy

Postmenopausal women with RA (N=49), aged 45–65 years, were included in a 2-year, randomized single-blinded controlled study,[29]. Patients had active disease with at least two of the following criteria: ≥ 6 painful joints, ≥ 3 swollen joints, erythrocyte sedimentation rate ≥ 20 mm per hour, and C-reactive protein ≥ 10 mg/l. Patients fulfilled the American Rheumatism Association 1987 criteria for RA,[30]. Women in the hormone replacement therapy (HRT) group were given continuous treatment with 2 mg E2 plus 1 mg norethisterone acetate daily. All patients gave their informed consent, and the Ethics Committee at the University of Gothenburg (Sweden) approved the study.

Serum measurements

In the sera from the human HRT study, ACPA were evaluated by ELISA (Orgentec Inc). In mice, IgGs and anti-OVA specific IgGs were measured by the commercial kits (Bethyl Laboratories). For OVA-specific IgG, plates were coated with 100µg/mL OVA (Sigma), incubated with sera (diluted 1:5000) and detected with HRP-conjugated polyclonal rabbit-anti mouse IgG (Dako). For evaluating the affinity properties of antibodies, potassium thiocyanate (Sigma) was added in various doses. For measuring sialic acid residues on IgG or OVA-specific IgG biotinylated Sambucus nigra lectin (Vector Laboratories) and streptavidin-HRP (R&D) was used as detection.

Isolation of OVA-specific antibodies

OVA-specific antibodies were captured from serum of OVA-immunized mice. Protein-G isolated total IgG was dialyzed in sodium phosphate dibasic and enriched over OVA-coupled Sepharose 4B beads (Sigma). washed with NaCl four times and eluted with lectine buffer. ELISA confirmed enrichment of OVA-specific IgG.

Mass spectrometry analysis for Fc glycans

For the analysis of Fc-glycosylation, the IgG eluates were subjected to tryptic digest by adding 600 ng tosyl-phenylalanyl-chloromethyl-ketone treated trypsin (Merck) in 40 µL ammonium bicarbonate buffer followed by overnight incubation at 37°C. Digested IgG was separated and analyzed on an Ultimate 3000 UHPLC system (Dionex Corporation) coupled to a Maxis Impact HD quadrupole-time-of-flight mass spectrometer (MS) (Bruker Daltonics). Details are described in the supplementary methods. Quality of mass spectra was evaluated based on total intensities per glycopeptide cluster. Analyte curation was performed using the signal-to-noise ratio, isotopic pattern quality, and observed *m/z* deviation as obtained after data (pre-)processing with LacyTools,[31]. Following extraction of tryptic glycopeptides by a C18 solid phase extraction trap column (Dionex Acclaim PepMap100), separation was achieved on Ascentis Express C18 nano-liquid chromatography (LC) column (Supelco) conditioned at 900 nL/min with 0.1% TFA (mobile phase A) after which the following gradient of mobile phase A and 95% acetonitrile (mobile phase B) was applied: 0min 3% B, 2 min 6% B, 4.5 min 18% B, 5 min 30% B, 7 min 30% B, 8 min 1% B and 11min 1% B. The UPLC was interfaced to the MS with a CaptiveSpray ESI source and nanoBooster (Bruker Daltonics). Mass spectra were recorded from *m/z* 550 to 1800 at a frequency of 1 Hz. Quadrupole ion energy and collision energy of the MS were set at 2 and 5 eV, respectively. The total analysis time per sample was 13 min. Detailed calculation are submitted as supplementary method.

Cell preparation and flow cytometry

Cell suspensions were obtained from spleen and bone marrow and stained for surface markers after erythrolysis. fixation and permeabilization (eBioscience). Analyses were done by Gallios Flow Cytometer (Beckman Coulter), Kaluza software (Beckman Coulter). The following fluorochrome- or biotin-conjugated anti-mouse antibodies and reagents were used: APC-conjugated anti-CD267 (TACI), FITC-conjugated anti-B220, FITC-conjugated anti-CD11b (all eBioscience), PE-conjugated anti-CD138 and PE-Cy7 conjugated anti-CD3, Pacific Blue F4/80, PE-Cy7 Ly6G, PE FcγRIII (CD16) (all Biolegend), APC conjugated: FcγRI (CD64), FcγRIIB and FcγRIV (self-made), Alexa Fluor 488-conjugated OVA-A488 (Thermo Fisher), anti-β-galactoside α2,6-sialyltransferase (ST6gal1) (C) (IBL) and normal rabbit IgG (isotype-matched control antibody) (Biotechnology).

Mouse B cell proliferation

Splenic B cells were isolated by CD43 depletion using MACS technology (Miltenyi). The cells were stimulated with LPS (Sigma) and cultured for 48 h to develop into plasmablasts. The medium was then changed to serum-free medium with no estrogen or 10^-8^M 17β-estradiol (E2) (Sigma) for the last 24h. Blocking antibodies towards IL22 (Biolegend Poly5164) and TNF-α (Ultra-LEAF anti-TNF-α; Biolegend MP6-XT22) were added. 72 h after initial seeding and 24 h after change in medium, supernatants were collected and cells were isolated for RNA-analyses.

Plasma cell isolation

CD138^+^ splenic plasmablasts were isolated using MACS technology (Miltenyi) from ovariectomized OVA-immunized mice, treated with estrogen or placebo. The purified cells were isolated for RNA- analyses.

Human B-cell proliferation

Human B cells were purified from peripheral blood mononuclear cells (PBMCs) using immunomagnetic beads (Dynal® B cell Negative Isolation Kit). The cells were stimulated with TLR-9 agonist cytosine–phosphate–guanine (CpG) oligodeoxynucleotide 2006 (InvivoGen), goat anti-human IgA/IgG/IgM F(ab’)_2_ fragments (Jackson ImmunoResearch Laboratories) and human recombinant IL-2 (R&D Systems), and cultured for 5 days to develop into plasmablasts. After 5 days medium was changed to no estrogen or 10^-8^M 17β-estradiol (E2) (Sigma). 7 days after initial seeding and 48 h after medium change, cells were isolated for RNA-analyses.

Real-time polymerase chain reaction (RT-PCR)

Total RNA was extracted using RNeasy kit (Qiagen) and transcribed into cDNA using oligo(dT) primers and MuLV reverse transcriptase (Roche). The quantitative real-time PCR was performed with SYBR Green I-dTTP (Eurogentec) or Applied Biosystems StepOnePlus^TM^ Real-time PCR systems (PE Applied Biosystems) using assays-on-demands primer and probe set. The gene-expression values were normalized to those of the control gene encoding β-actin and 18S. Primers sequences are described in the supplementary methods.

Statistical analysis

Statistical analyses were performed using GraphPad Prism (La Jolla). Two separate groups were compared with unpaired Student’s t-test. Several groups one-way ANOVA followed by Bonferroni multiple comparison tests were performed for selected columns. Outliers were eliminated using Grubbs’ test. The correlation was investigated with Spearman correlation coefficient. Data are presented as mean +/- standard error of the mean or as scatter plot, p<0.05 was considered significant.

Results

Effects of menopausal state and E2 treatment on antibody responses to ovalbumin immunization

To understand the effect of estrogen on the humoral immune response we immunized mice with ovalbumin (OVA). Prior to immunization, mice were either sham-operated or ovariectomized (OVX) with or without subsequent treatment with E2, reflecting hormone replacement therapy or estrogen deficiency, respectively. As expected, OVA immunization increased total IgG and OVA-specific IgG (Fig 1a and 1b). E2 treatment increased total IgG2b and IgG1 levels but no differences were found in the development of OVA-specific IgG suggesting that E2 does not influence the antibody titers after immunization. Also, the affinity of OVA-specific antibodies was not affected by estrogen status (Fig 1c).

Effects of menopausal state and E2 treatment on IgG sialylation

Estrogen treatment increased terminal sialic acid residues of total IgG (Fig 1d). Even more interesting, this increase was also seen in OVA-specific IgG (Fig 1e), while ovariectomy itself induced a significant decrease of sialylation of OVA-specific IgG. Mass spectrometric analysis of purified OVA-specific IgG2 showed significantly lower sialic acid content in ovariectomized mice, while E2 treatment rescued IgG2 sialylation (Fig 1f). In contrast, no difference was found in OVA-depleted IgG2, indicating that estrogen determines the sialylation of newly formed antigen-specific antibodies. Galactose, that is positioned proximal to sialic acids was much lesser affected by E2 (Fig 1f), although a trend (p=0.08) towards higher galactosylation was found in OVA-specific IgG2 after E2 treatment. Overall these results indicate that estrogen status regulates sialic acid content of newly formed IgG. Reversely, E2 deficiency suppresses the sialylation of IgGs. These observations support the hypothesis that E2 could regulate IgG-mediated effector functions.

Effects of menopausal state and E2 treatment on Fc gamma receptor (FcγR) expression

We next determined estrogens regulation of FcγR expression in OVA-challenged mice treated with E2. E2 status affects the B cell and plasma cell frequency, therefore the mean fluorescence intensity was used for determine the expression quantity of each FcγR. In the bone marrow, we observed that estrogen significantly increased the expression of the inhibitory FcγRIIb in plasmablasts, monocytes and neutrophils (Fig 2a). In the spleen, similar effects could only be observed with neutrophils (Fig 2b). Expression of activating FcγRIII and FcγRIV, were not affected by either ovariectomy or E2 treatment (Fig 2c-d). Furthermore, OVA-specific IgG subclass composition was not significantly affected by ovariectomy or E2 treatment, although a trend towards lower IgG2a and IgG2b levels was found after ovariectomy (Sup. Fig 1).

E2 increases the expression of β-galactoside α2,6-sialyltransferase 1 in plasmablasts

To explain the effects of E2 on IgG Fc sialylation, we analyzed the expression of β-galactoside α2,6-sialyltransferase 1 (St6gal1), the enzyme responsible for the attachment of sialic acid residues to IgG, in plasmablasts. mRNA was isolated from splenic plasmablasts purified from post-menopausal mice (OVX) with and without E2 treatment that had been challenged by OVA immunization. The identity of the plasmablasts was confirmed by measuring mRNA expression of the plasma cell-specific marker Blimp1 (Fig 3a). Expression of St6gal1 was significantly up-regulated by E2. In contrast, expression of β-1,4-galactosyltransferase 1 (B4galt1), which is responsible for IgG galactosylation, was hardly affected. St6gal1 protein expression of plasmablasts was further investigated using flow cytometry analysis. E2 treatment showed significantly higher expression of St6gal1 in total splenic plasmablasts (Fig 3b) as well as in OVA-specific plasmablasts (Fig 3c).

To determine whether E2 directly affects St6gal1 expression or whether this effect is mediated by cytokines, we stimulated naïve splenic B cells by lipopolysaccharide (LPS) to induce plasmablast differentiation. Blimp1 mRNA was up-regulated and IgG production was increased confirming plasmablast differentiation (Fig 3d). E2 treatment up-regulated IgG levels compared to E2 restriction. As expected, St6gal1 mRNA was significantly up-regulated upon E2 treatment, while no effect was seen for B4galt1 (Fig 3e). Cytokines such as TNFα, which are regulated by E2,[32], as well as IL-22 have been shown to influence IgG glycosylation [20, 33]. We therefore blocked TNFα and IL-22 to test whether cytokine inhibition influences the effect of E2 on ST6gal1 expression. However, up-regulation of St6gal1 was still evident after blocking with anti-TNF-α and anti-IL-22 antibodies (Fig 3e), suggesting that E2 up-regulation of St6gal1 is independent of TNF-α and IL-22.

E2 treatment in human RA patients increases antibody sialylation

To confirm the relevance of the above-described findings for patients with RA, we examined serum samples of 49 postmenopausal women with RA treated with or without hormone replacement therapy [29, 34, 35]. In a previous trial, HRT treatment was shown to reduce disease activity of RA and to increase bone mineral density. There was no difference in ACPA titers between patients receiving HRT or not receiving HRT (Fig 4a) with totally 7 ACPA negative patients, 3 in the HRT treated and 4 in the controls. For the following investigations, we concentrated on ACPA positive patients: IgG galactosylation and sialylation in the 42 ACPA-positive patients were increased in the HRT treated patients in both the Fc regions of IgG1 (Fig 4b-c) and IgG2/3 (Sup Fig 2a-b). Fucose residues were not altered in the Fc regions of IgG1 (Fig 4d) or IgG2/3 (Sup Fig 2c). These data indicate that E2 treatment increase sialic acid content on IgG-Fc-tails in postmenopausal women with RA. The increase in IgG sialylation and galactosylation in the individual patients over time is depicted in supplementary figure 3. Furthermore, when correlating the degree of IgG sialylation and galactosylation to serum E2 levels a significant positive correlation was found, adding further evidence that E2 can regulate effector function of IgGs in humans (Suppl Figure 4a and 4b). In addition, IgG sialylation and galactosylation showed a significant inverse correlation with RA disease activity as measured by DAS score [29] (Suppl Figure 4c and 4d).

E2 increases St6gal1 expression in human plasmablasts

Finally, and based on the data obtained from the human cohort, we analyzed whether E2 can induce St6gal1 expression in human plasmablasts. To test this, we isolated human B cells from healthy controls, differentiated them into plasmablasts and added E2 for the last 48h. E2 significantly up-regulated mRNA expression of St6gal1 in the human plasmablasts, while no such effects were observed for B4galt1 (Fig 4e). Hence, E2 induces St6gal1 and IgG sialylation also in humans.

Discussion

Herein, we show that E2 influences IgG glycosylation, especially the sialylation of IgG by up-regulating the key enzyme St6gal1 in plasmablasts. Deficiency of E2, like in the menopause, leads to decreased antibody sialylation and to a pro-inflammatory IgG pattern, which could influence the onset of RA and may explain the increased risk of RA in postmenopausal women. On the other hand, E2 administration increased sialylation of IgG shifting antibody effector function to a more regulatory anti-inflammatory pattern, which is supported by a negative correlation between the degree of IgG sialylation and galactosylation with RA disease activity.

In RA, it is well established that autoantibody formation precedes the symptomatic inflammatory phase of the disease. Factors that shift asymptomatic autoimmunity to inflammation are therefore of key interest in understanding the onset of disease,[36]. Sialylation status of autoantibodies seems to play a crucial role in this shift. Low-level IgG sialylation promotes progression to inflammation, while high-level sialylation promotes staying asymptomatic,[20]. Our results indicate that estrogen affects the pathogenicity of the antibodies mainly via regulation of IgG-Fc sialylation. Hence, higher levels of E2 create an anti-inflammatory environment by inducing St6gal1, resulting in higher degree of antibody sialylation. In accordance, the sharp decrease of estrogens in the menopause is supposed to switch this environment to low sialylation and a pro-inflammatory pattern.

In contrast to sialylation we did not detect any significant effect of E2 on galactosylation in mice, or on the expression of B4galt1, the enzyme mediating galactosylation, in mouse and human antibody producing cells. Nonetheless, in postmenopausal RA patients, treatment with E2 not only increased sialylation but also galactosylation of IgG. Similarly, we observed strong correlations of the IgG-Fc galactosylation and levels of estrogen in the postmenopausal RA patients. These findings are in accordance with previous results in healthy individuals showing that E2 regulates galactosylation,[26]. As galactosylation is a prerequisite for sialylation at Asn297 site of IgG, an effect of E2 on galactosylation might further strengthen the overall E2-induced glycosylation pattern of human IgG.

E2 effects on B cell [37-39] and plasma cell differentiation [40, 41] have been previously reported, but functional consequences on the pattern of plasma cell-mediated antibody production have so far been undetermined. Regulation of St6gal1 by E2 suggests that the overall effect of E2 on the effector pathways of adaptive immunity is a regulatory one and that loss of E2 induces a pro-inflammatory environment by altering effector functions of antibodies. On the other hand, E2 did not have any consistent effect on specific antibody levels and affinity, suggesting that the key factor, by which E2 regulates inflammatory responses, is indeed its influence on IgG glycosylation. Future studies, will need to test whether estrogen treatment in postmenopausal women stimulates St6gal1 in B cells and plasmablasts.

Conclusion

In conclusion, these data provide a molecular concept that could explain why susceptibility to RA changes during a woman’s life and specifically increases in the menopause. E2 appears to be a protective rather than a risk factor in triggering inflammation in arthritis by inhibiting the pro-inflammatory effector functions of autoantibodies. Higher rates of flares of RA with the decrease of sex hormones after pregnancy,[42] as well as the accumulation of flares in the second low-estrogen phase of the menstrual cycle,[43] additionally support this concept. Treatment with E2 may therefore have a beneficial effect in some RA patients, particularly in those with imminent RA displaying autoantibodies and initial symptoms, such as pain, with a high risk of progressing to clinical RA.

Abbreviations

Anti-citrullinated peptide antibodies (ACPA)

Estrogen (E2)

Fc gamma receptors (FcγR)

Hormone replacement therapy (HRT)

Lipopolysaccharide (LPS)

Mass spectrometer (MS)

Ovalbumin (OVA)

Ovariectomized (OVX)

Rheumatoid arthritis (RA)

β-galactoside α2,6-sialyltransferases 1 (St6gal1)

β-1,4-galactosyltransferase 1 (B4galt1)

Ethics approval and consent to participate

Animal studies was approved of the ethics committees at Government of Mittelfranken, Germany and University of Gothenburg, Sweden. All patients gave their informed consent, and the Ethics Committee at the University of Gothenburg (Sweden) approved the study.

Consent for publication

Not applicable

Availability of data and material

The dataset used and analyzed during the current study are available from the corresponding author on reasonable request.

Competing interests

The authors declare no competing financial interests.

Funding

This study was supported by the Swedish Research Council (537-2013-7370), Deutsche Forschungsgemeinschaft (SPP1468 Immunobone and CRC 1181), the Bundesministerium für Bildung und Forschung (BMBF; METARTHROS), the Marie-Curie project Osteoimmune, the TEAM project of the European Union, the IMI-funded project RTCure, the Swedish Association for Medical Research, the Interdisciplinary Centre for Clinical Research of the University of Erlangen-Nuremberg, Cancerfonden Konung Gustav V stiftelse and Åke Wiberg stiftelse.

Contributions

C.E. and G.S. design the study. C.E. J.R. U.H. R.P performed animal experiment, cell cultivation experiment and interpreted the data. A.B. performed mass spectrometry measurement, A.B. M.W and H.U.S interpreted data and provided expertise and input. A.C. and L.M performed the human cell cultivation interpreted data and provide expertise. M.S. and F.N provided the antibodies for Fc gamma receptors, interpreted data and provide expertise. H.F.D provided the postmenopausal RA patients, interpreted data and provide expertise. C.E. G.K. H.F.D and G.S. provided expertise and input in putting all data together. C.E. U.H. and G.S. wrote the manuscript. All authors critically reviewed and approved the final form of the manuscript.

Acknowledgements

We acknowledge the excellent assistance from Silke Winkler, Wolfgang Baum, Merja Nurkkula- Karlsson, Katharina Falk, Petra Henning and Marcus Söderberg. We also thank Holger Bang (Orgentec) for providing ACPA tests for the project.

References

1. Goemaere S, Ackerman C, Goethals K, De Keyser F, Van der Straeten C, Verbruggen G, Mielants H, Veys EM: **Onset of symptoms of rheumatoid arthritis in relation to age, sex and menopausal transition**. *The Journal of rheumatology* 1990, **17**(12):1620-1622.

2. Brennan P, Bankhead C, Silman A, Symmons D: **Oral contraceptives and rheumatoid arthritis: results from a primary care-based incident case-control study**. *Seminars in arthritis and rheumatism* 1997, **26**(6):817-823.

3. McInnes IB, Schett G: **The pathogenesis of rheumatoid arthritis**. *The New England journal of medicine* 2011, **365**(23):2205-2219.

4. Catrina AI, Svensson CI, Malmstrom V, Schett G, Klareskog L: **Mechanisms leading from systemic autoimmunity to joint-specific disease in rheumatoid arthritis**. *Nature reviews Rheumatology* 2017, **13**(2):79-86.

5. Malmstrom V, Catrina AI, Klareskog L: **The immunopathogenesis of seropositive rheumatoid arthritis: from triggering to targeting**. *Nature reviews* 2017, **17**(1):60-75.

6. Rantapaa-Dahlqvist S, de Jong BA, Berglin E, Hallmans G, Wadell G, Stenlund H, Sundin U, van Venrooij WJ: **Antibodies against cyclic citrullinated peptide and IgA rheumatoid factor predict the development of rheumatoid arthritis**. *Arthritis and rheumatism* 2003, **48**(10):2741-2749.

7. Berglin E, Padyukov L, Sundin U, Hallmans G, Stenlund H, Van Venrooij WJ, Klareskog L, Dahlqvist SR: **A combination of autoantibodies to cyclic citrullinated peptide (CCP) and HLA-DRB1 locus antigens is strongly associated with future onset of rheumatoid arthritis**. *Arthritis research & therapy* 2004, **6**(4):R303-308.

8. Svensson L, Jirholt J, Holmdahl R, Jansson L: **B cell-deficient mice do not develop type II collagen-induced arthritis (CIA)**. *Clinical and experimental immunology* 1998, **111**(3):521-526.

9. Kleinau S, Martinsson P, Heyman B: **Induction and suppression of collagen-induced arthritis is dependent on distinct fcgamma receptors**. *J Exp Med* 2000, **191**(9):1611-1616.

10. Holmdahl R, Rubin K, Klareskog L, Larsson E, Wigzell H: **Characterization of the antibody response in mice with type II collagen-induced arthritis, using monoclonal anti-type II collagen antibodies**. *Arthritis and rheumatism* 1986, **29**(3):400-410.

11. Nimmerjahn F, Ravetch JV: **Antibody-mediated modulation of immune responses**. *Immunological reviews* 2010, **236**:265-275.

12. Yamaguchi Y, Nishimura M, Nagano M, Yagi H, Sasakawa H, Uchida K, Shitara K, Kato K: **Glycoform-dependent conformational alteration of the Fc region of human immunoglobulin G1 as revealed by NMR spectroscopy**. *Biochimica et biophysica acta* 2006, **1760**(4):693-700.

13. Dekkers G, Treffers L, Plomp R, Bentlage AEH, de Boer M, Koeleman CAM, Lissenberg-Thunnissen SN, Visser R, Brouwer M, Mok JY *et al*: **Decoding the Human Immunoglobulin G-Glycan Repertoire Reveals a Spectrum of Fc-Receptor- and Complement-Mediated-Effector Activities**. *Frontiers in immunology* 2017, **8**:877.

14. Parekh RB, Dwek RA, Sutton BJ, Fernandes DL, Leung A, Stanworth D, Rademacher TW, Mizuochi T, Taniguchi T, Matsuta K *et al*: **Association of rheumatoid arthritis and primary osteoarthritis with changes in the glycosylation pattern of total serum IgG**. *Nature* 1985, **316**(6027):452-457.

15. Rademacher TW, Williams P, Dwek RA: **Agalactosyl glycoforms of IgG autoantibodies are pathogenic**. *Proceedings of the National Academy of Sciences of the United States of America* 1994, **91**(13):6123-6127.

16. Hess C, Winkler A, Lorenz AK, Holecska V, Blanchard V, Eiglmeier S, Schoen AL, Bitterling J, Stoehr AD, Petzold D *et al*: **T cell-independent B cell activation induces immunosuppressive sialylated IgG antibodies**. *The Journal of clinical investigation* 2013, **123**(9):3788-3796.

17. Ito K, Furukawa J, Yamada K, Tran NL, Shinohara Y, Izui S: **Lack of galactosylation enhances the pathogenic activity of IgG1 but Not IgG2a anti-erythrocyte autoantibodies**. *J Immunol* 2014, **192**(2):581-588.

18. Ohmi Y, Ise W, Harazono A, Takakura D, Fukuyama H, Baba Y, Narazaki M, Shoda H, Takahashi N, Ohkawa Y *et al*: **Sialylation converts arthritogenic IgG into inhibitors of collagen-induced arthritis**. *Nature communications* 2016, **7**:11205.

19. Bohm S, Schwab I, Lux A, Nimmerjahn F: **The role of sialic acid as a modulator of the anti-inflammatory activity of IgG**. *Semin Immunopathol* 2012, **34**(3):443-453.

20. Pfeifle R, Rothe T, Ipseiz N, Scherer HU, Culemann S, Harre U, Ackermann JA, Seefried M, Kleyer A, Uderhardt S *et al*: **Regulation of autoantibody activity by the IL-23-TH17 axis determines the onset of autoimmune disease**. *Nature immunology* 2016.

21. Rombouts Y, Ewing E, van de Stadt LA, Selman MH, Trouw LA, Deelder AM, Huizinga TW, Wuhrer M, van Schaardenburg D, Toes RE *et al*: **Anti-citrullinated protein antibodies acquire a pro-inflammatory Fc glycosylation phenotype prior to the onset of rheumatoid arthritis**. *Annals of the rheumatic diseases* 2015, **74**(1):234-241.

22. Chen G, Wang Y, Qiu L, Qin X, Liu H, Wang X, Wang Y, Song G, Li F, Guo Y *et al*: **Human IgG Fc-glycosylation profiling reveals associations with age, sex, female sex hormones and thyroid cancer**. *Journal of proteomics* 2012, **75**(10):2824-2834.

23. Bakovic MP, Selman MH, Hoffmann M, Rudan I, Campbell H, Deelder AM, Lauc G, Wuhrer M: **High-throughput IgG Fc N-glycosylation profiling by mass spectrometry of glycopeptides**. *J Proteome Res* 2013, **12**(2):821-831.

24. Bondt A, Selman MH, Deelder AM, Hazes JM, Willemsen SP, Wuhrer M, Dolhain RJ: **Association between galactosylation of immunoglobulin G and improvement of rheumatoid arthritis during pregnancy is independent of sialylation**. *J Proteome Res* 2013, **12**(10):4522-4531.

25. van de Geijn FE, Wuhrer M, Selman MH, Willemsen SP, de Man YA, Deelder AM, Hazes JM, Dolhain RJ: **Immunoglobulin G galactosylation and sialylation are associated with pregnancy-induced improvement of rheumatoid arthritis and the postpartum flare: results from a large prospective cohort study**. *Arthritis research & therapy* 2009, **11**(6):R193.

26. Ercan A, Kohrt WM, Cui J, Deane KD, Pezer M, Yu EW, Hausmann JS, Campbell H, Kaiser UB, Rudd PM *et al*: **Estrogens regulate glycosylation of IgG in women and men**. *JCI insight* 2017, **2**(4):e89703.

27. Nilsson ME, Vandenput L, Tivesten A, Norlen AK, Lagerquist MK, Windahl SH, Borjesson AE, Farman HH, Poutanen M, Benrick A *et al*: **Measurement of a Comprehensive Sex Steroid Profile in Rodent Serum by High-Sensitive Gas Chromatography-Tandem Mass Spectrometry**. *Endocrinology* 2015, **156**(7):2492-2502.

28. Offner H, Adlard K, Zamora A, Vandenbark AA: **Estrogen potentiates treatment with T-cell receptor protein of female mice with experimental encephalomyelitis**. *The Journal of clinical investigation* 2000, **105**(10):1465-1472.

29. D'Elia HF, Larsen A, Mattsson LA, Waltbrand E, Kvist G, Mellstrom D, Saxne T, Ohlsson C, Nordborg E, Carlsten H: **Influence of hormone replacement therapy on disease progression and bone mineral density in rheumatoid arthritis**. *The Journal of rheumatology* 2003, **30**(7):1456-1463.

30. Arnett FC, Edworthy SM, Bloch DA, McShane DJ, Fries JF, Cooper NS, Healey LA, Kaplan SR, Liang MH, Luthra HS *et al*: **The American Rheumatism Association 1987 revised criteria for the classification of rheumatoid arthritis**. *Arthritis and rheumatism* 1988, **31**(3):315-324.

31. Jansen BC, Falck D, de Haan N, Hipgrave Ederveen AL, Razdorov G, Lauc G, Wuhrer M: **LaCyTools: A Targeted Liquid Chromatography-Mass Spectrometry Data Processing Package for Relative Quantitation of Glycopeptides**. *J Proteome Res* 2016, **15**(7):2198-2210.

32. Ralston SH, Russell RG, Gowen M: **Estrogen inhibits release of tumor necrosis factor from peripheral blood mononuclear cells in postmenopausal women**. *J Bone Miner Res* 1990, **5**(9):983-988.

33. Croce A, Firuzi O, Altieri F, Eufemi M, Agostino R, Priori R, Bombardieri M, Alessandri C, Valesini G, Saso L: **Effect of infliximab on the glycosylation of IgG of patients with rheumatoid arthritis**. *J Clin Lab Anal* 2007, **21**(5):303-314.

34. D'Elia HF, Mattsson LA, Ohlsson C, Nordborg E, Carlsten H: **Hormone replacement therapy in rheumatoid arthritis is associated with lower serum levels of soluble IL-6 receptor and higher insulin-like growth factor 1**. *Arthritis research & therapy* 2003, **5**(4):R202-209.

35. d'Elia HF, Carlsten H: **The impact of hormone replacement therapy on humoral and cell-mediated immune responses in vivo in post-menopausal women with rheumatoid arthritis**. *Scand J Immunol* 2008, **68**(6):661-667.

36. McInnes IB, Schett G: **Pathogenetic insights from the treatment of rheumatoid arthritis**. *Lancet* 2017, **389**(10086):2328-2337.

37. Masuzawa T, Miyaura C, Onoe Y, Kusano K, Ohta H, Nozawa S, Suda T: **Estrogen deficiency stimulates B lymphopoiesis in mouse bone marrow**. *The Journal of clinical investigation* 1994, **94**(3):1090-1097.

38. Erlandsson MC, Jonsson CA, Islander U, Ohlsson C, Carlsten H: **Oestrogen receptor specificity in oestradiol-mediated effects on B lymphopoiesis and immunoglobulin production in male mice**. *Immunology* 2003, **108**(3):346-351.

39. Hill L, Jeganathan V, Chinnasamy P, Grimaldi C, Diamond B: **Differential roles of estrogen receptors alpha and beta in control of B-cell maturation and selection**. *Molecular medicine (Cambridge, Mass)* 2011, **17**(3-4):211-220.

40. Verthelyi DI, Ahmed SA: **Estrogen increases the number of plasmablasts and enhances their autoantibody production in nonautoimmune C57BL/6 mice**. *Cellular immunology* 1998, **189**(2):125-134.

41. Grimaldi CM, Jeganathan V, Diamond B: **Hormonal regulation of B cell development: 17 beta-estradiol impairs negative selection of high-affinity DNA-reactive B cells at more than one developmental checkpoint**. *J Immunol* 2006, **176**(5):2703-2710.

42. Ostensen M, Aune B, Husby G: **Effect of pregnancy and hormonal changes on the activity of rheumatoid arthritis**. *Scand J Rheumatol* 1983, **12**(2):69-72.

43. Colangelo K, Haig S, Bonner A, Zelenietz C, Pope J: **Self-reported flaring varies during the menstrual cycle in systemic lupus erythematosus compared with rheumatoid arthritis and fibromyalgia**. *Rheumatology (Oxford, England)* 2011, **50**(4):703-708.

Figure Legends

**Figure 1. Estrogen influences IgG sialylation.**

Mice were ovariectomized at 3 months of age, followed by insertion of slow-release treatment pellets with placebo (Pla) or estrogen (E2; 0.83 μg/day). 10 days after ovariectomy the animals were immunized with ovalbumin (OVA) and 14 days later boostered. Serum was taken day 9 (d9) before immunization, day 22 (d22) after immunization, and day 38 (d38) at termination after immunization and boostering. (**a)** Total IgG concentrations: Significant induction in all treatment groups, after immunization (d9-d22) and after boostering (d22-d38) for all IgG subtypes. Data are mean ±SEM (n=11-13). (**b)** Concentration of ovalbumin-specific IgG (OVA-IgG) (n=17-24). (**c)** Titers of ovalbumin-specific IgG (n=10-12). (**d)** Concentration of sialic acids on total IgG (n=17-24). (**e)** Concentration of sialic acids on OVA-specific IgG (n=17-24). (**f)** Mass-spectrometry-based analysis of sialic acids and galactose on IgG-Fc of OVA-specific (OVA-spec) and OVA depleted (OVA-dep) IgG2 (n=12-14). Values are indicated as scatter dot plot with mean indicated with a bar. Statistical analysis with ANOVA followed by Bonferroni multiple comparisons for selected time point *p<0.05, ***p<0.001.

**Figure 2. Effects of estrogen on Fc gamma receptor expression**

Ovariectomized mice at three months of age followed by insertion of slow-release treatment pellets with placebo (Pla) or estrogen (E2; 0.83 μg/day). 10 days after ovariectomy the animals were immunized with ovalbumin (OVA) and 14 days later boostered. On day 38, mean fluorescence intensity (MFI) of Fc receptors (FcR) was quantifed using flow cytometry in all leucocytes as well as B cells, plasmablasts, monocytes and neutrophils. Expression of inhibitory FcRIIb in (**a)** bone marrow cells and (**b)** spleen cells. Expression of stimulatory FcRIII and FcRIV, in (**c)** bone marrow cells and (**d)** spleen cells. Groups are presented with a bar indicating mean ±SEM (n=6-7). Statistical analysis with ANOVA followed by Bonferroni multiple comparisons for selected time point *p<0.05, ***p<0.001.

**Figure 3. Estrogen affects expression of the sialylation enzyme in plasmablasts.**

Mice were ovariectomized at 3 months of age, followed by insertion of slow-release treatment pellets with placebo (Pla) or estrogen (E2; 0.83 μg/day). 10 days after ovariectomy animals were immunized with ovalbumin (OVA) and 14 days later boostered. (**a)** Expression of Blimp1, β-1,4-galactosyltransferase 1 (B4galt1) and β-galactoside α2,6-sialyltransferase 1 (St6gal1) in CD138 sorted plasmablasts on day 38 (n=5-6). Statistical analysis with students t-test. (**b)** Flow cytometry based quantification of mean fluorescence intensity (MFI) of St6Gal1 expression of plasmablasts on day 38 (n=10-14). Statistical analysis with ANOVA followed by Bonferroni multiple comparisons. (**c)** Flow cytometry based quantification of MFI St6Gal1 expression of OVA-specific plasmablasts (n=10-14). Statistical analysis with ANOVA followed by Bonferroni multiple comparisons (**d)** IgG in supernatant and expression of Blimp1, B4galt1, and St6gal1 in CD43 negative splenic cells stimulated with LPS for 72 hours for developing into plasmablasts. The last 24 hours the cells were stimulated with the presence (+) of estrogen (E2) 10^-8^ or absence (-) of E2. Anti-TNFα or ant-IL-22 antibodies were also added as indicated. Statistical analysis with students t-test (n=5-7). *p<0.05**p<0.01 Data are shown as mean ±SEM.

**Figure 4. Estrogen increases IgG glycosylation in postmenopausal RA patients.**

49 postmenopausal women with active rheumatoid arthritis (RA) included in a randomized controlled trial receiving either hormone replacement therapy (HRT) or no HRT. Serum samples were taken at baseline, after 1 year and 2 years of treatment. (**a)** Anti-citrullinated proteins antibody concentrations (n=20-29), 7 anti-CCP negative patients were excluded from further studies). Mass-spectrometry-based analysis of (**b)** sialic acids, (**c)** galactose and (**d)** fucose on the Fc portion of IgG. Statistical analysis with t-test at selected time points, as well as an investigation over time in HRT, treated individuals *p<0.05, ***p<0.001 (n=17-25). Groups are presented with a bar indicating mean with SEM (**e)** RNA expression of St6gal1 and B4galt1 from B cells isolated from peripheral blood collected from healthy donors and stimulated with CpG, IL-2 and goat anti-human IgA/IgG/IgM F(ab’)_2_ fragments for 5 days to induce plasmablasts differentiation. The last 48 hours the cells were stimulated with the presence (+) of estrogen (E2) 10^-8^ or absence (-) of E2. Statistical analysis with students t-test *p<0.05 (n=3). Values are indicated as scatter dot plot with means indicated with a bar.

Supplementary Figure Legends

**Supplementary Figure 1. Estrogen effects on ovalbumin-specific IgG subclasses.** Ovariectomized mice received slow-release treatment pellets with placebo (Pla) or estrogen (E2; 0.83 μg/day). 10 days after ovariectomy the animals were immunized with ovalbumin (OVA) and 14 days later boostered. On day 38, IgG subclasses of ovalbumin (OVA)-specific IgG were measured. Scatter plot with means indicated by lines; N=9-11 mice per group). Statistical analysis performed with ANOVA followed by Bonferroni multiple comparisons.

**Supplementary Figure 2. Estrogen treatment increases IgG-Fc sialylation and IgG-Fc galactosylation in postmenopausal rheumatoid arthritis patients.** 49 postmenopausal women with active rheumatoid arthritis (RA) included in a randomized controlled trial receiving either hormone replacement therapy (HRT) or no HRT. Serum samples were taken at baseline, after 1 year and 2 years of treatment. Mass-spectrometry-based analysis of (**a)** sialic acids, (**b)** galactose and (**c)** fucose of the Fc portion of IgG2/3 (n=17-25). Statistical analysis with t-test at selected time points, as well as an investigation over time in HRT treated individuals *p<0.05, ***p<0.001.

**Supplementary Figure 3. Effects of estrogen on IgG-Fc sialylation and IgG-Fc galactosylation in individual patients**

Analysis of IgG-Fc sialylation in **(a, b)** and IgG-Fc galactosylation **(c, d)** in postmenopausal women with active rheumatoid arthritis (RA) randomized to receive hormone replacement therapy (HRT) (**a, c**) or no HRT (**b, d**) in a controlled trial. Each line represents one patient.

**Supplementary Figure 4. Correlation between IgG glycosylation and estrogen levels and disease activity**

Correlation between (**a)** IgG-Fc sialylation and (**b)** IgG-Fc galactosylation (y-axis) and estrogen level (x-axis). Correlation between (**c)** IgG-Fc sialylation and (**d)** IgG-Fc galactosylation (y-axis) and disease activity score (DAS) (x-axis). Spearman´s correlation coefficients (r) and p- values are shown.
