# Supplementary material for: Estrogen induces St6gal1 expression and increases IgG sialylation in mice and patients with rheumatoid arthritis: a potential explanation for the increased risk of rheumatoid arthritis in postmenopausal women
Source: Arthritis Res Ther. 2018 May 2;20:84. doi: 10.1186/s13075-018-1586-z (PMC5932893; doi:10.1186/s13075-018-1586-z)
Supplement: Supplementary file 2 — Supplementary Figure 1. Estrogen effects on ovalbumin-specific IgG subclasses. Ovariectomized mice received slow-release treatment pellets with placebo (Pla) or estrogen (E2; 0.83 μg/day). Ten days after ovariectomy, the animals were immunized with ovalbumin (OVA), and 14 days later, they were boostered. On day 38, IgG subclasses of ovalbumin (OVA)-specific IgG were measured. A scatterplot with means indicated by lines is shown (n = 9–11 mice per group). Statistical analysis was performed with analysis of variance followed by the Bonferroni multiple-comparisons test. (PDF 1088 kb) [file 13075_2018_1586_MOESM2_ESM.pdf]

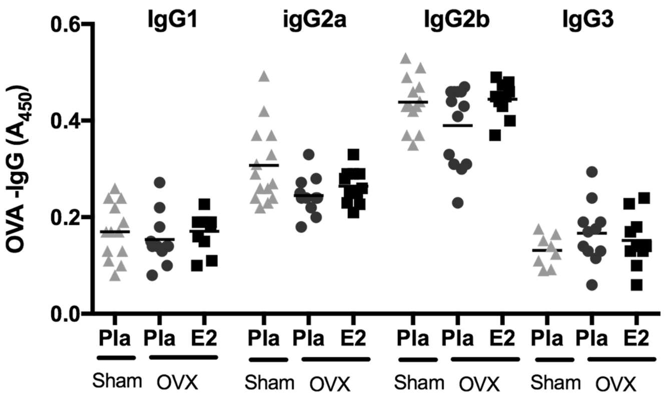

Supplementary Figure 1. Estrogen effects on ovalbumin-specific IgG subclasses. Ovariectomized mice received slow-release treatment pellets with placebo (Pla) or estrogen (E2; 0.83 µg/day). Ten days after ovariectomy, the animals were immunized with ovalbumin (OVA), and 14 days later, they were boostered. On day 38, IgG subclasses of ovalbumin (OVA)-specific IgG were measured. A scatterplot with means indicated by lines is shown (*n* = 9–11 mice per group). Statistical analysis was performed with analysis of variance followed by the Bonferroni multiple-comparisons test.

Additional file 3:
