# Supplementary material for: Estrogen induces St6gal1 expression and increases IgG sialylation in mice and patients with rheumatoid arthritis: a potential explanation for the increased risk of rheumatoid arthritis in postmenopausal women
Source: Arthritis Res Ther. 2018 May 2;20:84. doi: 10.1186/s13075-018-1586-z (PMC5932893; doi:10.1186/s13075-018-1586-z)
Supplement: Supplementary file 3 — Supplementary Figure 2. Estrogen treatment increases IgG-Fc sialylation and IgG-Fc galactosylation in postmenopausal patients with rheumatoid arthritis (RA). Forty-nine postmenopausal women with active RA were included in a randomized controlled trial receiving either hormone replacement therapy (HRT) or no HRT. Serum samples were taken at baseline and after 1 year and 2 years of treatment. Mass spectrometry-based analysis of (a) sialic acids, (b) galactose, and (c) fucose of the Fc portion of IgG2/3 (n = 17–25). Statistical analysis was performed with t tests at selected time points, as well as in an investigation over time in individuals who received HRT. * p < 0.05, *** p < 0.001. (PDF 1915 kb) [file 13075_2018_1586_MOESM3_ESM.pdf]

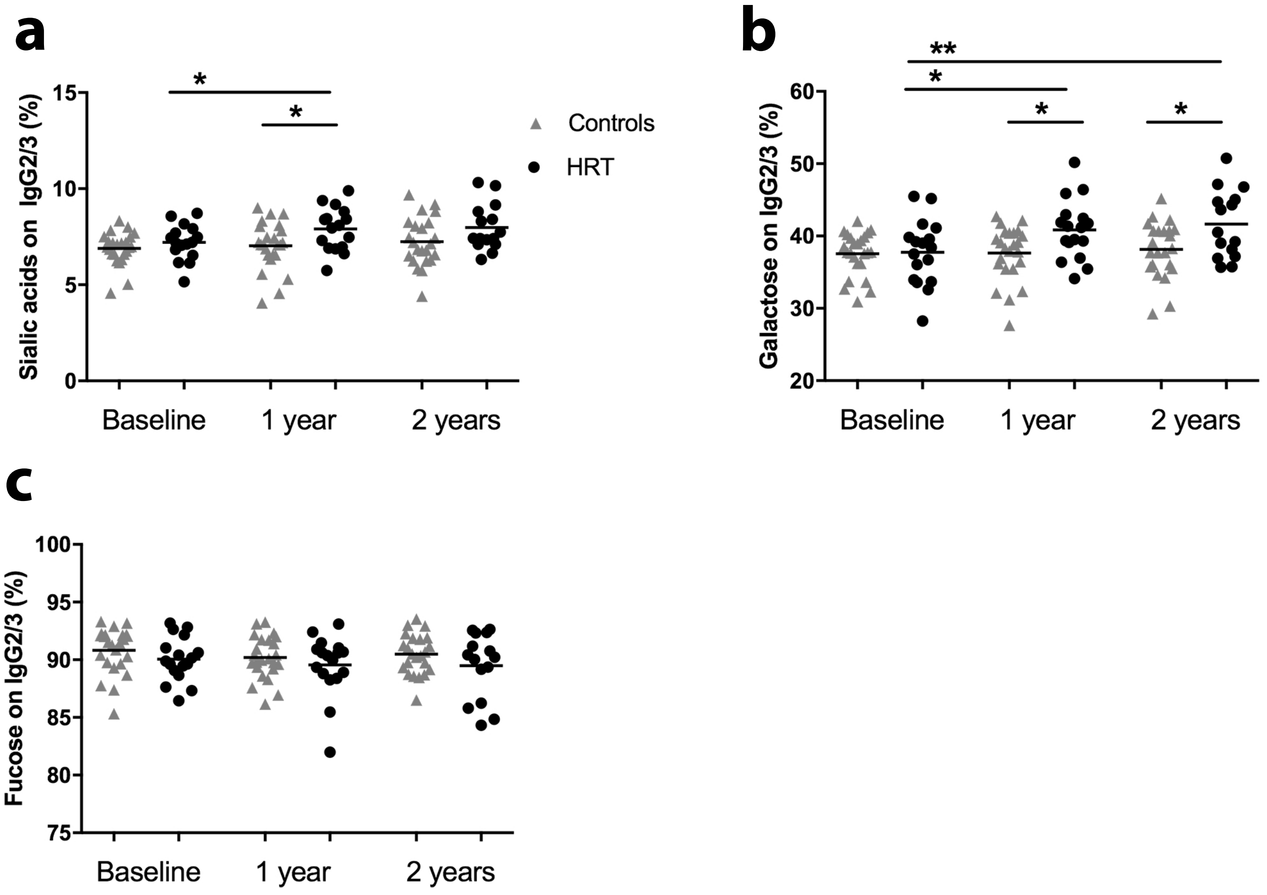

Supplementary Figure 2. Estrogen treatment increases IgG-Fc sialylation and IgG-Fc galactosylation in postmenopausal patients with rheumatoid arthritis (RA). Forty-nine postmenopausal women with active RA were included in a randomized controlled trial receiving either hormone replacement therapy (HRT) or no HRT. Serum samples were taken at baseline and after 1 year and 2 years of treatment. Mass spectrometry-based analysis of (**a**) sialic acids, (**b**) galactose, and (**c**) fucose of the Fc portion of IgG2/3 (n = 17–25). Statistical analysis was performed with t tests at selected time points, as well as in an investigation over time in individuals who received HRT. * $p < 0.05$, *** $p < 0.001$.
