# Supplementary material for: Estrogen induces St6gal1 expression and increases IgG sialylation in mice and patients with rheumatoid arthritis: a potential explanation for the increased risk of rheumatoid arthritis in postmenopausal women
Source: Arthritis Res Ther. 2018 May 2;20:84. doi: 10.1186/s13075-018-1586-z (PMC5932893; doi:10.1186/s13075-018-1586-z)
Supplement: Supplementary file 4 — Supplementary Figure 3. Effects of estrogen on IgG-Fc sialylation and IgG-Fc galactosylation in individual patients. Analysis of (a, b) IgG-Fc sialylation and (c, d) IgG-Fc galactosylation in postmenopausal women with active rheumatoid arthritis (RA) randomized to receive hormone replacement therapy (HRT) (a, c) or no HRT (b, d) in a controlled trial. Each line represents one patient. (PDF 2784 kb) [file 13075_2018_1586_MOESM4_ESM.pdf]

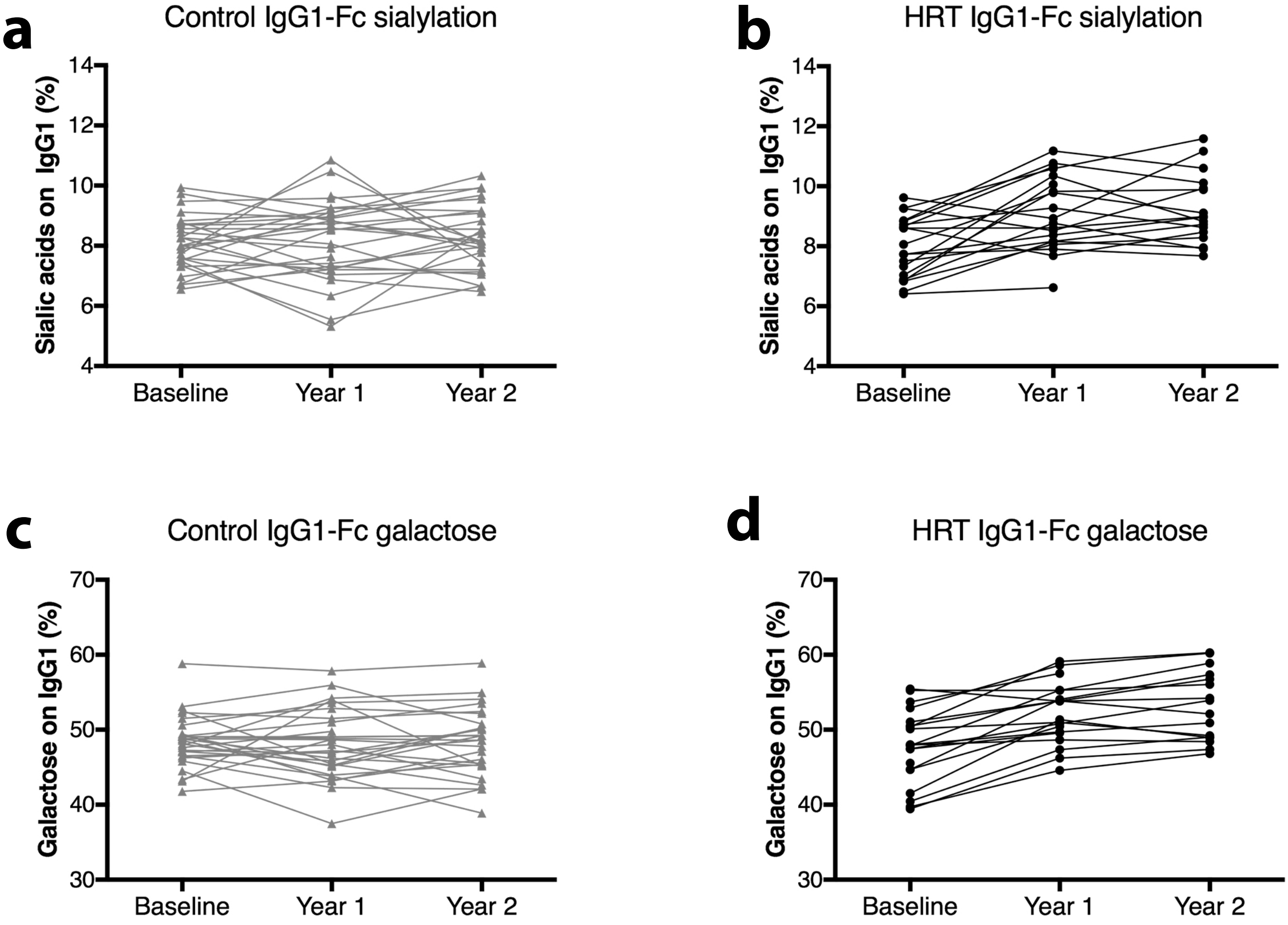

Supplementary Figure 3. Effects of estrogen on IgG-Fc sialylation and IgG-Fc galactosylation in individual patients. Analysis of (**a**, **b**) IgG-Fc sialylation and (**c**, **d**) IgG-Fc galactosylation in postmenopausal women with active rheumatoid arthritis (RA) randomized to receive hormone replacement therapy (HRT) (**a**, **c**) or no HRT (**b**, **d**) in a controlled trial. Each line represents one patient.
