# Supplementary material for: Estrogen induces St6gal1 expression and increases IgG sialylation in mice and patients with rheumatoid arthritis: a potential explanation for the increased risk of rheumatoid arthritis in postmenopausal women
Source: Arthritis Res Ther. 2018 May 2;20:84. doi: 10.1186/s13075-018-1586-z (PMC5932893; doi:10.1186/s13075-018-1586-z)
Supplement: Supplementary file 5 — Supplementary Figure 4. Correlation between IgG glycosylation and estrogen levels and disease activity. Correlation between (a) IgG-Fc sialylation and (b) IgG-Fc galactosylation (y-axis) and estrogen level (x-axis). Correlation between (c) IgG-Fc sialylation and (d) IgG-Fc galactosylation (y-axis) and Disease Activity Score (DAS) (x-axis). Spearman’s correlation coefficients (r) and p values are shown. (PDF 2188 kb) [file 13075_2018_1586_MOESM5_ESM.pdf]

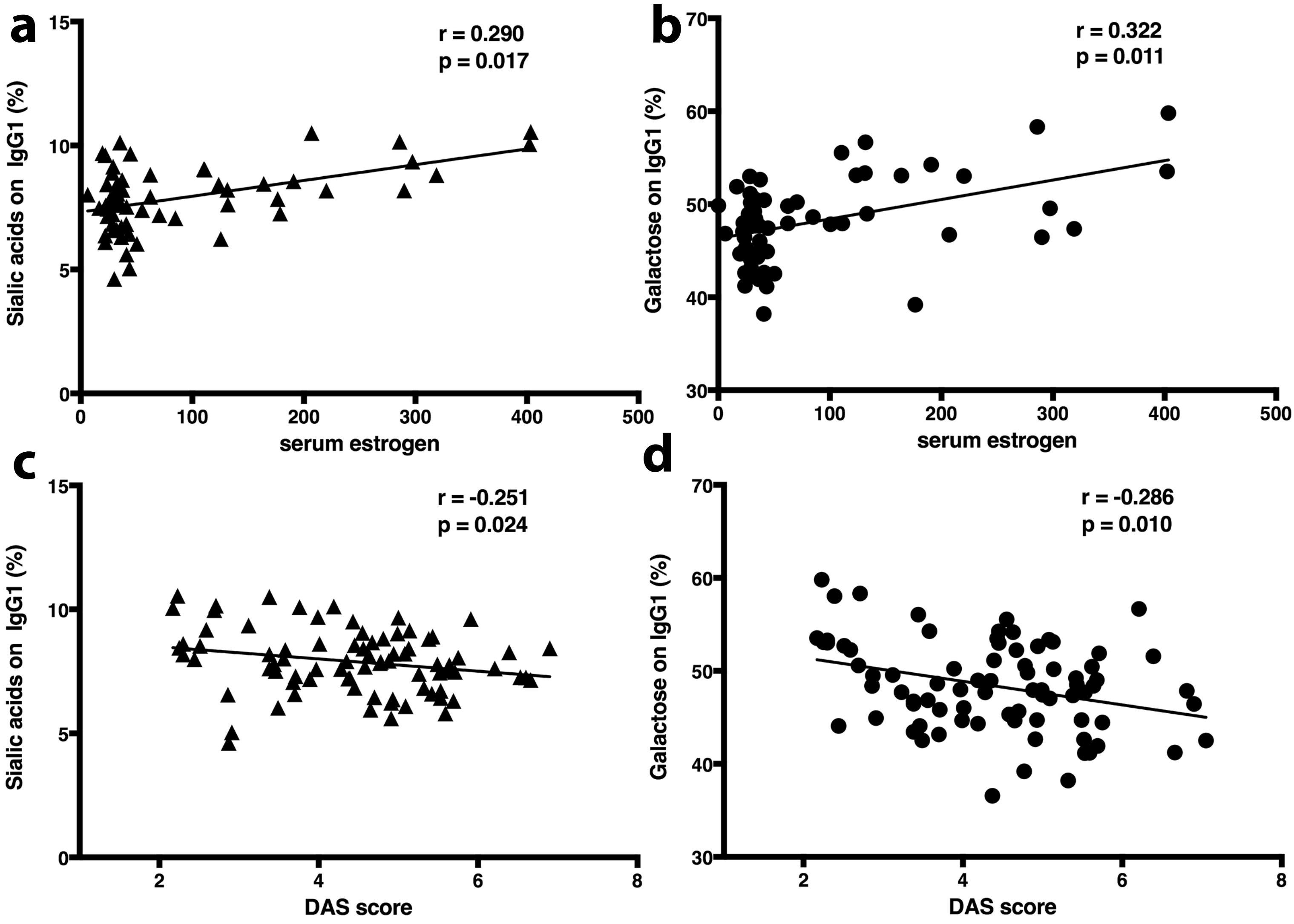

Supplementary Figure 4. Correlation between IgG glycosylation and estrogen levels and disease activity. Correlation between (**a**) IgG-Fc sialylation and (**b**) IgG-Fc galactosylation (*y*-axis) and estrogen level (*x*-axis). Correlation between (**c**) IgG-Fc sialylation and (**d**) IgG-Fc galactosylation (*y*-axis) and Disease Activity Score (DAS) (*x*-axis). Spearman's correlation coefficients (*r*) and *p* values are shown.
